# Development and validation of a duplex droplet digital PCR assay for the simultaneous detection of cytomegalovirus and Epstein-Barr virus in plasma

**DOI:** 10.3389/fcimb.2026.1798127

**Published:** 2026-03-19

**Authors:** Zhaoxiang Du, Xingxing Yuan, Sihan Zhou, Lili Zhang, Yifeng Wang, Jie Yi, Manyu Li, Yan Dang, Ning Liu, Xin Liu, Fangfang Dai, Haiqing Sun, Yanhua Yu, Gengxia Yang

**Affiliations:** 1 Department of Clinical Laboratory Center, Beijing Youan Hospital, Capital Medical University, Beijing, China; 2 Department of Clinical Laboratory, Peking Union Medical College Hospital, Chinese Academy of Medical Sciences, Beijing, China; 3 Division I of In Vitro Diagnostics for Infectious Diseases, Institute for In Vitro Diagnostics Control, National Institutes for Food and Drug Control, Beijing, China; 4 Department of General Surgery, Beijing Youan Hospital, Capital Medical University, Beijing, China

**Keywords:** CMV, coinfection, ddPCR, DNAemia, EBV, molecular assays, qPCR

## Abstract

**Background:**

Human cytomegalovirus (CMV) and Epstein-Barr virus (EBV) are globally prevalent herpesviruses. While typically self-limiting in immunocompetent individuals, infections can cause severe consequences even in this population. In immunocompromised groups, such as transplant recipients and HIV-infected individuals, viral reactivation or coinfection frequently triggers graft rejection, multi-organ invasion, and malignancies, often exhibiting synergistic pathogenicity. Current serological assays are limited by “window periods” and delayed immune responses, while traditional quantitative PCR (qPCR) relies on standard curves for quantification. Consequently, there is an urgent need for precise, interference-resistant methods. This study aimed to develop and validate a duplex droplet digital PCR (ddPCR) assay for the simultaneous, absolute quantification of CMV and EBV in plasma.

**Methods:**

Based on the TD-1 platform, a single-tube duplex detection system targeting conserved viral regions was optimized to minimize the “rain effect” and maximize signal-to-noise ratios. Leveraging the absolute quantification capability of ddPCR without standard curves, we compared its performance, including dynamic range and limit of detection (LOD), against a homologous qPCR assay. Clinical validation was conducted using 117 plasma samples from suspected cases, utilizing a commercial qPCR kit as the reference standard. Additionally, tolerance to endogenous interfering substances was assessed.

**Results:**

The optimized duplex ddPCR demonstrated high analytical sensitivity, with LODs for CMV and EBV at 7.9 and 6.5 copies/reaction, respectively, which were approximately 6- to 7-fold lower than homologous qPCR (53.4 and 45.6 copies/reaction).No competitive inhibition was observed at low concentrations. In clinical validation, the assay achieved 100% sensitivity and specificity compared to the reference kit, with high quantitative correlation (R^2^ = 0.80–0.87). Notably, ddPCR detected four weak positive samples (1 CMV, 3 EBV) missed by homologous qPCR. Furthermore, the method maintained accurate quantification in plasma containing hyperlipidemia or hyperbilirubinemia.

**Conclusion:**

This study successfully established a duplex ddPCR assay characterized by high sensitivity, specificity, and robust interference resistance. By enabling precise absolute quantification, it serves as a powerful complement to existing technologies for the early diagnosis and monitoring of CMV and EBV active infections.

## 1 Introduction

Human cytomegalovirus (CMV, HHV-5) and Epstein-Barr virus (EBV, HHV-4) are two ubiquitous herpesviruses that infect a substantial proportion of the global population. As pervasive pathogens, primary infection typically occurs early in life, resulting in extremely high seroprevalence worldwide. In immunocompetent individuals, infections are generally asymptomatic or self-limiting, leading to the establishment of lifelong latency within the host (Nowalk et al., 2016; Zuhair et al., 2019). However, in immunocompromised populations, including solid organ transplant (SOT) and hematopoietic stem cell transplant (HSCT) recipients, as well as HIV-infected individuals, primary infection or reactivation of latent viruses often precipitates severe clinical consequences. CMV can invade multiple organ systems, including the central nervous system and lungs, and is closely associated with both acute and chronic graft injury and rejection (Dioverti et al., 2016). Conversely, EBV infection is a key driver of malignancies such as post-transplant lymphoproliferative disorder (PTLD), Hodgkin lymphoma, and nasopharyngeal carcinoma (Chakravorty et al., 2022).

Notably, given their overlapping epidemiology and host immune status, coinfection or dual reactivation of CMV and EBV is clinically common and often manifests synergistic pathogenic effects. Studies have demonstrated that in HSCT and SOT recipients, concurrent activation of both viruses significantly increases the risk of viral pneumonia, hemorrhagic cystitis, and PTLD, while also elevating graft rejection rates, thereby markedly reducing one-year overall survival (Anderson‐Smits et al., 2020; Zhou et al., 2021; Li et al., 2022). Similarly, in HIV-infected patients and those undergoing immunosuppressive therapy, dual infection poses a grave threat to patient health and prognosis (Kato et al., 2022; Nazim et al., 2022). Given the formidable clinical challenges presented by CMV and EBV coinfection, simultaneous and precise quantitative monitoring of both viruses is critical for guiding preemptive clinical therapy.

In the laboratory diagnosis of CMV and EBV, traditional serological testing plays a significant role but is hampered by notable limitations. The inherent “window period” for antibody production can delay diagnosis and treatment. Furthermore, IgM antibodies may persist for months after infection resolution or reappear during reactivation, complicating the distinction between latent and active infection. Most critically, immunocompromised patients often exhibit impaired humoral immunity, leading to delayed or insufficient antibody responses, which creates a substantial risk of missed diagnosis. Consequently, serology is ill-suited for real-time diagnosis in these populations. In contrast, molecular assays directly reflect active viral replication by quantifying viral nucleic acid copy numbers (Dioverti et al., 2016; Abusalah et al., 2020). Beyond offering superior sensitivity and specificity, molecular methods are broadly applicable to diverse specimen types, including whole blood, plasma, cerebrospinal fluid, and bronchoalveolar lavage fluid. Crucially, they provide precise viral load data that accurately mirror *in vivo* replication kinetics, which is essential for monitoring antiviral efficacy, assessing disease progression risk, and guiding early intervention (Guiver et al., 2001; Piiparinen et al., 2004).

Since the invention of polymerase chain reaction (PCR) in 1985, molecular diagnostics has evolved over four decades from first-generation end-point PCR and second-generation real-time quantitative PCR (qPCR) to the emerging third-generation technology: digital PCR (dPCR). As the prevailing standard, qPCR relies on relative quantification via standard curves and reference materials, precluding the direct acquisition of absolute target copy numbers. This methodology is heavily dependent on operator skill and the accuracy of standards, with results susceptible to variations in curve fitting and batch-to-batch inconsistencies. Moreover, qPCR exhibits limited sensitivity and precision for low-copy samples and is vulnerable to inhibition by complex matrix components, potentially leading to underestimation or false negatives (Nyaruaba et al., 2022). Conversely, dPCR partitions a reaction into thousands of independent micro-units, each theoretically containing zero or one template molecule. Post-amplification, target copy numbers are calculated directly using Poisson statistics based on the proportion of positive units. This approach enables absolute quantification without standard curves. The compartmentalized design and end-point data acquisition of dPCR confer significant advantages in detecting low-abundance templates, tolerating PCR inhibitors, and maintaining high sensitivity in complex backgrounds (Cao et al., 2017; Kojabad et al., 2021). Depending on the partitioning method, dPCR platforms are primarily categorized into chip-based dPCR (cdPCR) and droplet-based dPCR (ddPCR). While cdPCR isolates nanoliter-scale reactions in micro-wells or channels, ddPCR utilizes water-in-oil emulsion technology to generate tens of thousands of droplets. Compared to cdPCR, ddPCR ensures physical and chemical isolation of micro-reactors with minimal cross-contamination and easier enumeration. Furthermore, as cdPCR relies on complex microfluidic chip fabrication, ddPCR offers superior cost-effectiveness and scalability, demonstrating greater potential for clinical application. Currently, this technology has been validated in critical scenarios such as ultrasensitive detection of infectious diseases and screening for rare cancer mutations (Xu et al., 2023). Recently, Professor Yong Guo’s team at Tsinghua University developed a low-cost microdroplet generation chip based on thermoplastic polycarbonate, achieving stable monodisperse droplet formation (Su et al., 2019). Subsequently, the team engineered a high-sensitivity droplet reader based on laser-induced fluorescence cytometry. By optimizing the confocal optical path to eliminate defocus noise and significantly enhance the signal-to-noise ratio, combined with a novel contamination-free puncture-based microfluidic interface, this system enables automated, contamination-free, and high-throughput absolute quantification of fluorescence signals within droplets post-amplification (Zhu et al., 2020).

During the active phase of primary infection or reactivation, the lytic replication of CMV and EBV within host cells releases viral genomic DNA into the peripheral circulation, resulting in DNAemia. Unlike latent intracellular viral DNA, the presence and concentration of cell-free viral DNA in plasma are considered the most direct biomarkers of active viral replication *in vivo* (Tan et al., 2020; Damania et al., 2022; Aydın Güçlü et al., 2024; Cevenini et al., 2025). Addressing these clinical needs and leveraging the proprietary ddPCR platform described above, this study aims to develop and validate a duplex ddPCR assay for the simultaneous detection of CMV and EBV in plasma. We established a single-tube duplex system through optimized primer and probe design and systematically evaluated its dynamic range, sensitivity, specificity, and resistance to interference. Furthermore, using plasma samples from clinically suspected cases, we compared the duplex ddPCR results with those from a routine singleplex qPCR assay to verify its clinical value in reducing sample consumption and improving the detection rate of low-viral-load samples, thereby facilitating the precise diagnosis of CMV and EBV infections.

## 2 Materials and methods

### 2.1 Primer/probe design and preparation of standard controls

To ensure high sensitivity and broad coverage, the *UL54* gene of CMV (encoding DNA polymerase) and the *EBNA-1* gene of EBV (encoding nuclear antigen 1) were selected as amplification targets. The coding DNA sequences (CDS) of these reference genes were retrieved from the National Center for Biotechnology Information (NCBI, https://www.ncbi.nlm.nih.gov/). Specific primers and TaqMan probes were designed using the Primer3Plus online software (http://www.primer3plus.com/) following standard design principles. All primers and probes were synthesized by Sangon Biotech (Shanghai, China), and their specific sequences are listed in **Supplementary Table 1**. Primer specificity was confirmed via conventional PCR amplification and agarose gel electrophoresis to verify that the product size met expectations. Subsequently, PCR products were purified, cloned into the pUCm-T vector, and Sanger sequenced by Sangon Biotech. The final sequencing results were aligned with reference sequences in the NCBI database to confirm the accuracy of the amplified fragment sequences.

Two types of standard controls were prepared for different validation purposes. First, given the limited concentration of commercial viral DNA, recombinant plasmid standards were constructed to evaluate the linear range of the assay, particularly for high concentration intervals (>10^5^ copies/µL). Target gene fragments (*UL54* and *EBNA-1*) were chemically synthesized and cloned into the pUC57 vector (Biomed Gene Technology Co., Ltd., Beijing, China). Detailed sequence information and plasmid maps are provided in **Supplementary Figures 1a, b**. After determining the plasmid DNA concentration, the copy number was calculated using the following formula: DNA copy number (copies/µL) = [6.02 × 10^23^ × plasmid concentration (ng/µL) × 10^-9^]/[plasmid length (nt) × 660], where 6.02 × 10^23^ represents the Avogadro constant and 660 is the average molecular weight of a base pair (g/mol). Second, commercial inactivated viral DNA standards (CMV: ATCC VR-1780; EBV: ATCC VR-602) were utilized for method optimization and the assessment of the limit of detection (LOD), limit of quantification (LOQ), precision, and anti-interference capability to better simulate clinical samples.

### 2.2 Setup of the duplex ddPCR assay

**Figure 1:**
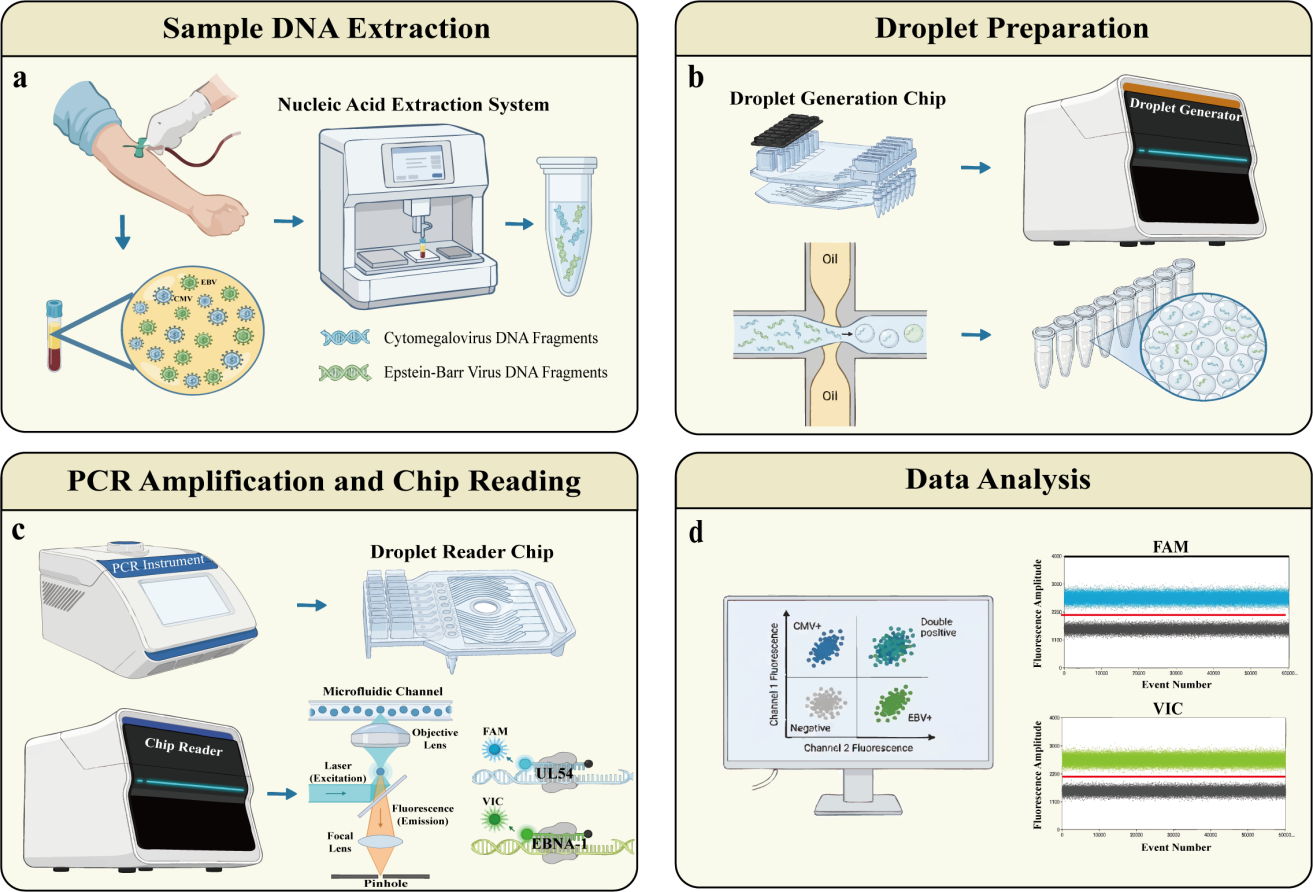
Schematic workflow of the duplex ddPCR assay for simultaneous detection of CMV and EBV. The assay procedure consists of four main steps. **(a)** Sample DNA Extraction. Viral genomic DNA is extracted from plasma separated from peripheral blood samples. **(b)** Droplet Preparation. The PCR reaction mixture and generation oil are loaded onto a microfluidic chip. Water-in-oil droplets are generated using the droplet generator. **(c)** PCR Amplification and Chip Reading. After thermal cycling, droplets are transferred to a detection chip. The chip reader detects fluorescence signals as droplets pass through the microfluidic channel (FAM channel for CMV UL54, VIC channel for EBV EBNA-1). **(d)** Data Analysis. Positive and negative droplet populations are distinguished based on fluorescence amplitude, and absolute copy numbers are calculated using Poisson statistics.

This study utilized the TD-1 droplet digital PCR platform (TargetingOne, Beijing, China). The workflow comprised four main steps: droplet generation, PCR amplification, droplet detection, and data analysis. Droplet generation and detection were performed using the Drop Maker M1 and Chip Reader R1, respectively (workflow illustrated in **Figures 1a–d**).

According to the manufacturer’s instructions, a 30 µL ddPCR reaction mixture was prepared, consisting of 7.5 µL of 4× PCR Master Mix (containing UNG), 1 µL of DNA template, varying volumes of primer and probe mix (depending on optimization results), and nuclease-free water. The 30 µL reaction mixture was loaded into the sample well of a droplet generation chip, and 180 µL of droplet generation oil was added to the oil well. After sealing with a specialized gasket, the chip was placed in the Drop Maker M1 to generate water-in-oil droplets, which were collected in reaction tubes. PCR amplification was performed on a thermal cycler with the following conditions: pre-denaturation at 95 °C for 30 s, followed by 40 cycles of denaturation at 94 °C for 10 s and annealing/extension at 56 °C–60 °C (specific temperature optimization described below). Upon completion of amplification, droplets were transferred to a detection chip. Driven by a pneumatic system, droplets were distributed into microchannels and passed sequentially through the detection zone for fluorescence excitation and reading. Finally, the absolute initial copy number of target molecules was calculated based on the Poisson distribution principle.

To establish an optimal duplex detection system, primer and probe concentrations as well as annealing temperatures were systematically optimized. First, primer and probe concentrations for CMV and EBV were screened separately in singleplex reactions. Various combinations of primer concentrations (400, 600, 800, 1000 nM) and probe concentrations (200, 300, 400, 500 nM) were tested. The optimal combination was selected based on criteria including clear separation between negative and positive droplet clusters, minimal “rain effect” (droplets with intermediate fluorescence intensity), and tight clustering of positive droplets. Once optimal concentrations were determined, the singleplex systems were combined into a duplex ddPCR system. Finally, the optimal annealing temperature for the duplex system was determined via a thermal gradient experiment (55 °C–60 °C).

### 2.3 Homologous qPCR assay protocol

To benchmark the analytical performance of the ddPCR assay against conventional quantitative PCR under identical conditions, a homologous qPCR system was established using the same primers, probes, and reaction system. The qPCR reaction had a total volume of 20 µL, containing 10 µL of 2× Premix Ex Taq (Takara Bio Inc., Shiga, Japan), 1.6 µL each of forward and reverse primers (final concentration: 800 nM), 0.6 µL each of probes (final concentration: 300 nM), 1 µL of DNA template, and 5.2 µL of nuclease-free water. Amplification and detection were performed on the Gentier 96E Real-Time PCR System (Tianlong Science and Technology, Xi’an, China). Thermal cycling conditions were set as follows: pre-denaturation at 95 °C for 30 s, followed by 40 cycles of denaturation at 94 °C for 10 s and annealing/extension at 58 °C for 30 s. Fluorescence signals were acquired at the end of each extension step.

### 2.4 Evaluation of dynamic range, analytical sensitivity and LOQ

Analytical performance was evaluated following the CLSI EP05-A2 guidelines, MIQE guidelines, and the updated Digital MIQE guidelines (Wittwer et al., 2009; Whale et al., 2020). To assess the dynamic range, recombinant plasmid standards (constructed as described in Section 2.1) were serially diluted. The linearity of the duplex ddPCR method was evaluated using plasmid templates ranging from 10^0^ to 10^5^ copies/µL. For comparison, the homologous qPCR method was evaluated over a broader range from 10^0^ to 10^6^ copies/µL. Analytical sensitivity was assessed by determining the limit of blank (LOB) and LOD. First, to calculate the LOB, 20 replicates of the no-template control (NTC) were tested. The LOB was defined as the copy number corresponding to the mean of the 20 NTC results plus three times the standard deviation (Mean_NTC_ + 3SD). Subsequently, to determine the LOD, commercial inactivated viral DNA standards were diluted to a series of low concentrations near the detection limit. For the ddPCR method, CMV was tested at 67, 30, 16, 10, 5, 3, and 1 copies/µL, and EBV was tested at 64, 37, 19, 11, 6, 4, and 1 copies/µL. For the homologous qPCR method, CMV was tested at 408, 209, 105, 57, 36, and 2 copies/µL, and EBV was tested at 470, 265, 85, 46, 15, and 8 copies/µL. Each concentration level was tested in 20 replicates. The LOD was calculated using Probit regression analysis (95% probability). Similarly, using 20 replicates of the viral DNA standards at the aforementioned concentrations, the lowest concentration with a coefficient of variation (CV) ≤ 20% was defined as the LOQ (Cao et al., 2025).

### 2.5 Assessment of competitive inhibition in duplex assay

Potential competitive inhibition between CMV and EBV primer/probe sets in the duplex system was assessed using commercial inactivated viral DNA standards. Two concentration levels were selected for evaluation: 10^2^ copies/µL (medium-low concentration) and 10^1^ copies/µL (low concentration). Samples were analyzed in both singleplex (detecting a single target only) and duplex (detecting both targets simultaneously) modes. Four replicates were performed for each condition to ensure data reliability. The impact of multiplexing on analytical performance was quantified by calculating the recovery rate using the following formula: Recovery Rate (%) = (Mean_Duplex_/Mean_Singleplex_) × 100%. A recovery rate falling within the range of 80%–120% was considered the criterion for demonstrating the absence of significant competitive inhibition between the CMV and EBV amplification systems.

### 2.6 Evaluation of analytical specificity

The analytical specificity of the duplex ddPCR system was evaluated against a panel of non-target microorganism samples, covering pathogens with genetic homology to the target viruses, those causing similar clinical symptoms, or those frequently causing bloodstream coinfections. Nucleic acid extraction was performed according to pathogen type: genomic DNA from bacterial and fungal strains was extracted using a Bacterial Genomic DNA Extraction Kit (Sevice Biology, Wuhan, China), while viral genomes were extracted using a Viral Genome Extraction Kit (Tiangen Biotech, Beijing, China). Specifically, input concentrations were standardized based on sample type to ensure rigorous specificity testing. For bacterial and fungal reference strains, genomic DNA was extracted from approximately 10^7^ cells and normalized to a concentration of 50 ng/µL. For clinical positive QCs, standardized samples with a concentration of 1.0×10^4^ copies/mL were utilized. For retrospective positive clinical specimens (e.g., HSV-1, VZV), nucleic acid extracts were obtained from partner laboratories (PUMCH, NIFDC) where they had been confirmed positive with viral loads exceeding the LOD of their respective clinical diagnostic assays. All extracted samples were tested using the optimized duplex ddPCR system, with inactivated CMV and EBV viral DNA standards serving as positive controls.

### 2.7 Assessment of precision

The precision of the duplex ddPCR system was verified by evaluating repeatability (intra-assay precision) and reproducibility (inter-assay precision). The experiment utilized commercial inactivated viral DNA standards at two concentration levels as templates: 10^2^ copies/µL and 10^1^ copies/µL. In the repeatability study, eight replicates of each concentration level were tested by the same operator in a single run. In the reproducibility study, eight replicates were performed separately by two different operators. Detection performance was analyzed by calculating the CV and recovery rate. Acceptance criteria for verifying repeatability and reproducibility were defined as a CV ≤ 20% and a recovery rate within the range of 80%–120%.

### 2.8 Plasma viral nucleic acid extraction

Viral nucleic acids were extracted from plasma samples utilizing the commercial magnetic bead-based Ex-DNA/RNA Virus 4.0 Kit in conjunction with the GeneRotex96 Nucleic Acid Extraction System (Tianlong Science and Technology, Xi’an, China), strictly following the manufacturer’s instructions. Briefly, 250 µL of plasma sample and 20 µL of Proteinase K were added to pre-packaged reagent plates. The automated nucleic acid extraction instrument utilized specialized magnetic rods to adsorb, transfer, and release magnetic beads, thereby achieving automated isolation and purification of nucleic acids. The final purified viral DNA was eluted in 80 µL of elution buffer and served as the template for subsequent ddPCR and qPCR testing.

### 2.9 Clinical sample collection and testing

A total of 117 residual nucleic acid extracts from clinical testing of patients with suspected CMV or EBV infection were collected for this study. These nucleic acid samples were prepared according to the clinical laboratory’s standard protocol, extracting from 250 µL of plasma and eluting in 80 µL of elution buffer. The results from a commercial CMV/EBV nucleic acid detection kit (DaAn Gene, Guangzhou, China) routinely used in the clinical laboratory served as the reference standard. This kit is approved by the National Medical Products Administration (NMPA) of China and is widely used in clinical practice. According to the manufacturer’s rigorous internal quality control standards and validation data, the kit maintains a LOD of 500 copies/mL (plasma).

To evaluate the clinical performance of the ddPCR method, these residual clinical nucleic acid extracts were tested using the established duplex ddPCR method and its homologous qPCR method. Notably, during the clinical sample testing phase, the template input volume for both ddPCR and homologous qPCR was set to 5 µL to enhance detection capabilities for samples with low viral loads. Since the LOD of the commercial kit is defined in copies/mL (plasma), whereas the raw output data for duplex ddPCR and homologous qPCR are in copies/reaction, the analytical LOD obtained in the method validation (see Section 3.2) and the quantitative results of clinical samples were converted to copies/mL for direct comparison. This conversion accounted for the input-to-elution volume ratio of nucleic acid extraction (250 µL input/80 µL elution) and the template loading volume of the PCR reaction. Clinical sensitivity and specificity were subsequently calculated and analyzed using the commercial kit results as the reference.

### 2.10 Evaluation of anti-interference capability against endogenous substances

To evaluate the tolerance of the established duplex ddPCR system to these complex clinical matrices, plasma samples from patients diagnosed with hyperlipidemia or hyperbilirubinemia were retrospectively screened and collected from the clinical case database; only samples confirmed negative for both CMV and EBV nucleic acids were selected. After nucleic acid extraction, inactivated viral DNA standards were added to the extracts to a final concentration near the LOD level. Simultaneously, ddH_2_O spiked with the same concentration of standards served as the control group. Four replicates were performed for each group. Quantitative results indicated no statistically significant difference in detection values between the hyperlipidemia/hyperbilirubinemia groups and the control group, even under high lipid and bilirubin backgrounds (*p*>0.05). Recovery rates were calculated using the formula: (Mean_Interference_/Mean_Control_) × 100%.

### 2.11 Statistical analysis

Data analysis and plotting were performed using GraphPad Prism 8.00 (GraphPad Software, La Jolla, CA, USA). Descriptive statistics were used to summarize data, including mean, standard deviation (SD), and CV. Linear regression analysis was employed to evaluate the dynamic range and linearity of the detection methods, with the coefficient of determination (R^2^) calculated to assess goodness of fit. The LOD was determined via Probit regression analysis (95% detection rate) using R software (R Foundation for Statistical Computing, Vienna, Austria). Comparisons of quantitative results between duplex ddPCR and homologous qPCR methods, as well as comparisons of recovery rates in interference experiments, were analyzed using paired t-tests where appropriate. All statistical tests were two-tailed, and *p* < 0.05 was considered statistically significant.

## 3 Results

### 3.1 Establishment and optimization of the duplex ddPCR assay

To construct a highly sensitive and specific detection system, highly conserved regions within the CMV and EBV genomes were initially screened as targets. The *UL54* gene of CMV and the *EBNA-1* gene of EBV were selected. Previous studies have confirmed that these gene regions exhibit extremely high conservation across different viral subtypes and demonstrate excellent performance as targets for clinical viral load monitoring (Habbal et al., 2009; Lay et al., 2010; Bilenoğlu et al., 2015; Ligozzi et al., 2016; Zealiyas et al., 2023). Specific primers and TaqMan probes were designed based on these target sequences. To verify primer effectiveness and specificity, conventional PCR amplification was performed using inactivated viral DNA standards as templates. Agarose gel electrophoresis revealed single, bright bands for both CMV and EBV amplification products, with sizes consistent with the expected fragment lengths (as shown in **Supplementary Figure 2**). Subsequently, to further confirm sequence accuracy, purified PCR products were TA-cloned, and positive clones were selected for Sanger sequencing. Sequence alignment showed 100% identity with the CMV *UL54* and EBV *EBNA-1* reference sequences in the NCBI database (as shown in **Supplementary Figures 3a, b**). These results confirmed that the designed primers and probes possessed excellent specificity suitable for the subsequent construction and optimization of the duplex ddPCR system.

To achieve optimal amplification efficiency and clear droplet partitioning, key reaction parameters of the duplex ddPCR system were systematically optimized. Gradients of primer and probe concentrations, as well as annealing temperatures, were evaluated, focusing on performance in the FAM (for CMV) and VIC (for EBV) fluorescence channels. The optimization process relied on two core evaluation criteria: the fluorescence amplitude and cluster tightness of positive droplets, and the separation between negative and positive droplet clusters to minimize the “rain effect.”

**Figure 2:**
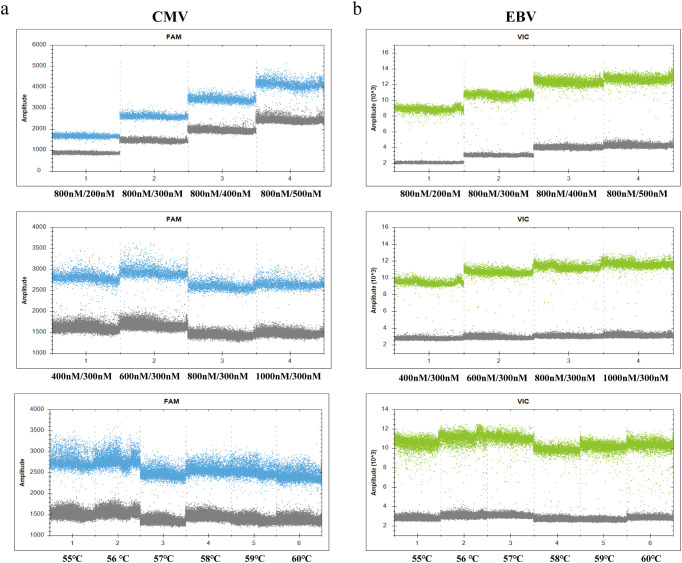
Optimization of reaction conditions for the duplex ddPCR assay. One-dimensional fluorescence amplitude plots showing the optimization results for **(a)** CMV (FAM channel) and **(b)** EBV (VIC channel). The top row displays probe concentration optimization performed with fixed primer concentrations at a constant annealing temperature of 59°C. The middle row shows primer concentration optimization using the fixed optimal probe concentration, also maintained at 59°C. The bottom row presents annealing temperature optimization (55°C–60°C) using the selected optimal primer and probe concentrations. Based on the cluster separation and signal amplitude, the final optimal conditions were determined to be 800 nM primers and 300 nM probes at 58°C. All experiments were performed in triplicate.

A stepwise optimization strategy was adopted to determine the optimal reaction system. Experimental results showed significant differences in droplet fluorescence patterns with adjustments to primer/probe concentrations and temperature. First, with primer concentration and annealing temperature fixed, the amplification effect of different probe concentration gradients was tested. Results indicated that a probe concentration of 300 nM for both CMV and EBV provided the best signal-to-noise ratio. Subsequently, primer concentrations were optimized based on the selected probe concentration; the strongest fluorescence signal and highest amplification efficiency were observed when CMV and EBV primer concentrations were both 800 nM. Finally, with fixed probe and primer concentrations, annealing temperatures ranging from 56 °C to 60 °C were tested. At 58 °C, the separation between droplets in both channels was optimal, with minimal “rain” interference. Based on these results, the optimal conditions for the duplex detection system were determined as follows: 800 nM for primers, 300 nM for probes, and an annealing temperature of 58 °C (as shown in **Figures 2a, b**; **Supplementary Figure 4**).

### 3.2 Comparative evaluation of dynamic range and sensitivity between duplex ddPCR and homologous qPCR

To systematically evaluate the analytical performance of the established duplex ddPCR system, a homologous qPCR method based on the same reaction system was constructed for head-to-head comparison. This qPCR method utilized identical primers, probe sequences, reaction concentrations, and annealing temperatures to eliminate biases caused by reaction condition differences and solely compare the performance of the two technology platforms.

**Figure 3:**
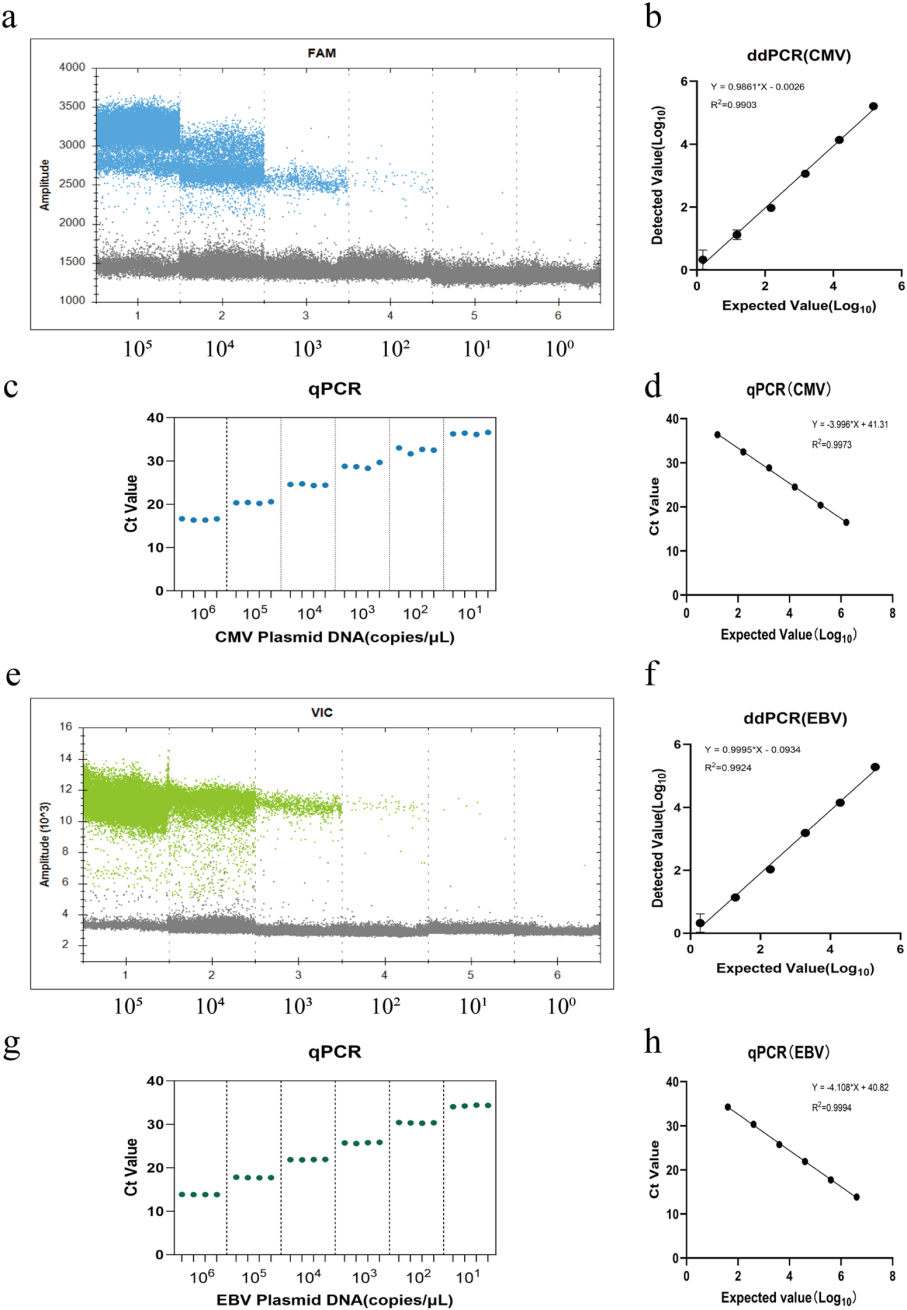
Comparison of linearity and dynamic range between duplex ddPCR and qPCR assays. Serial dilutions of plasmid standards were used to evaluate the analytical performance of both methods. **(a–d)** Analysis of CMV: **(a)** 1D fluorescence amplitude plot of ddPCR. **(b)** Linear regression analysis of the ddPCR assay, showing a dynamic range of 10^0^–10^5^ copies/μL(R^2^ = 0.9903). **(c)** Dot plot of qPCR Ct values. **(d)** Linear regression analysis of the qPCR assay, showing a dynamic range of 10^1^–10^6^ copies/μL(R^2^ = 0.9973). (e–h) Analysis of EBV: **(e)** 1D fluorescence amplitude plot of ddPCR. **(f)** Linear regression analysis of the ddPCR assay,showing a dynamic range of 10^0^–10^5^ copies/μL(R^2^ = 0.9924). **(g)** Dot plot of qPCR Ct values. **(h)** Linear regression analysis of the qPCR assay,showing a dynamic range of 10^1^–10^6^ copies/μL(R^2^ = 0.9994). The regression curves plot the log10-transformed expected copy numbers (x-axis) against the log10-transformed detected copy numbers (for ddPCR) or Ct values (for qPCR). All experiments were performed in triplicate.

The dynamic range of both methods was investigated using serially diluted CMV and EBV plasmids (concentration range: 10^0–^10^6^ copies/µL). Results showed that the ddPCR method exhibited a good linear relationship (R^2^>0.99) for CMV DNA and EBV DNA detection within the range of 10^0–^10^5^ copies/µL (as shown in **Figures 3a–d**). In contrast, the dynamic range for the qPCR method was 10^1–^10^6^ copies/µL (R^2^>0.99) (as shown in **Figures 3e–h**). The difference in detection ranges reflects the technical characteristics of each method. The upper quantitative limit of ddPCR is constrained by the total number of droplets generated. When the template concentration exceeds 3.0×10^5^ copies/µL, most droplets contain template molecules, causing the proportion of positive droplets to saturate and rendering Poisson statistics invalid; thus, its upper dynamic range limit is slightly lower than that of qPCR. However, in the low concentration range, ddPCR demonstrated significant advantages.

Before sensitivity assessment, to exclude background signal interference and ensure accuracy, positive criteria for both methods were established. For the duplex ddPCR system, 20 replicates of NTC samples were tested to determine the LOB. Statistical results showed mean background signals of 0.57 copies/reaction (SD = 1.08) for the FAM channel (CMV) and 0.38 copies/reaction (SD = 0.87) for the VIC channel (EBV). Based on the principle of LOB = Mean_NTC_ + 3 × SD_NTC_, the positive threshold for ddPCR was set at detection values higher than 3.8 copies/reaction (CMV) and 3.0 copies/reaction (EBV) (as shown in **Supplementary Figures 5a, b**). For the homologous qPCR system, considering differences in fluorescence channels and primer/probe combinations, target-specific thresholds were established. Specifically, 20 replicates of NTCs were tested, all of which yielded “Undetermined” results. Furthermore, we evaluated the amplification plots of standards near the expected detection limit; these low-level positive samples consistently exhibited distinct sigmoid (“S-shaped”) amplification curves. Based on this comprehensive assessment of curve morphology and the maximum valid Ct values observed in these standards, the positive threshold was set at Ct ≤ 37 for CMV and Ct ≤ 35 for EBV to exclude potential late-cycle non-specific background.

**Figure 4:**
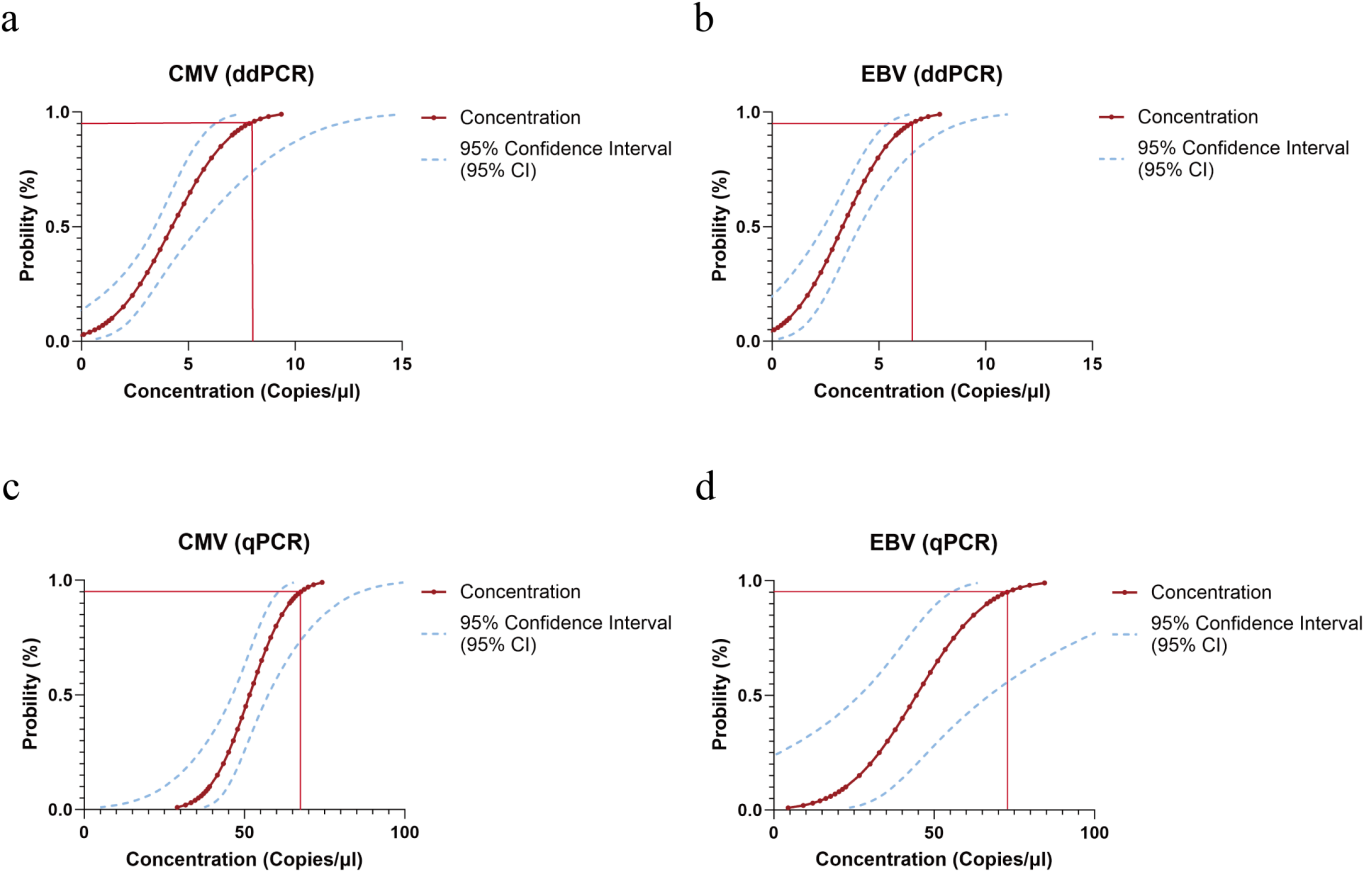
Probit regression analysis for determining the Limit of Detection (LOD). The detection probability was plotted against the viral DNA concentration to determine the 95% LOD. Each concentration point was tested in 20 replicates. **(a)** ddPCR assay for CMV: LOD determined at 7.9 copies/reaction. **(b)** ddPCR assay for EBV: LOD determined at 6.5 copies/reaction. **(c)** qPCR assay for CMV: LOD determined at 53.4 copies/reaction. **(d)** qPCR assay for EBV: LOD determined at 45.6 copies/reaction.The solid red lines represent the fitted probit regression curves. The blue dashed lines indicate the 95% confidence intervals (CI). The vertical red lines mark the concentration corresponding to a 95% detection probability.

Subsequently, to precisely determine the LOD of both methods, a series of concentration gradients of inactivated viral nucleic acid standards were prepared. Each concentration was tested in 20 replicates, and the concentration corresponding to a 95% detection probability was calculated using Probit regression analysis. Analysis indicated that the LODs of the duplex ddPCR system for CMV and EBV were as low as 7.9 copies/reaction (95% CI: 6.3–12.1) and 6.5 copies/reaction (95% CI: 5.4–8.8), respectively (as shown in **Figures 4a, b**). In comparison, the LODs for the homologous qPCR method were 53.4 copies/reaction (95% CI: 47.8–68.0) and 45.6 copies/reaction (95% CI: 38.3–58.8) (as shown in **Figures 4c, d**). These results demonstrate that the sensitivity of the constructed duplex ddPCR method is approximately 6 to 7 times that of the homologous qPCR method, confirming its significant advantage in detecting low-viral-load samples. Following LOD confirmation, the LOQ was further evaluated, defined as the lowest template concentration with a coefficient of variation (CV) less than 20% in 20 replicates. Experimental results determined the LOQs of the duplex ddPCR system for CMV and EBV as 9.9 copies/reaction and 10.8 copies/reaction, respectively. In contrast, the LOQs for the homologous qPCR method were 57 copies/reaction and 46 copies/reaction (as shown in **Supplementary Table 2**). This indicates that the duplex ddPCR system maintains good repeatability at low copy number levels, with its LOQ highly close to its LOD, achieving detection-as-quantification performance. Combined with the dynamic range results, the reliable quantitative range of the duplex ddPCR system was established as 9.9 to 10^5^ copies/reaction for CMV and 10.8 to 10^5^ copies/reaction for EBV.

### 3.3 Assessment of competitive inhibition in duplex assay

**Figure 5:**
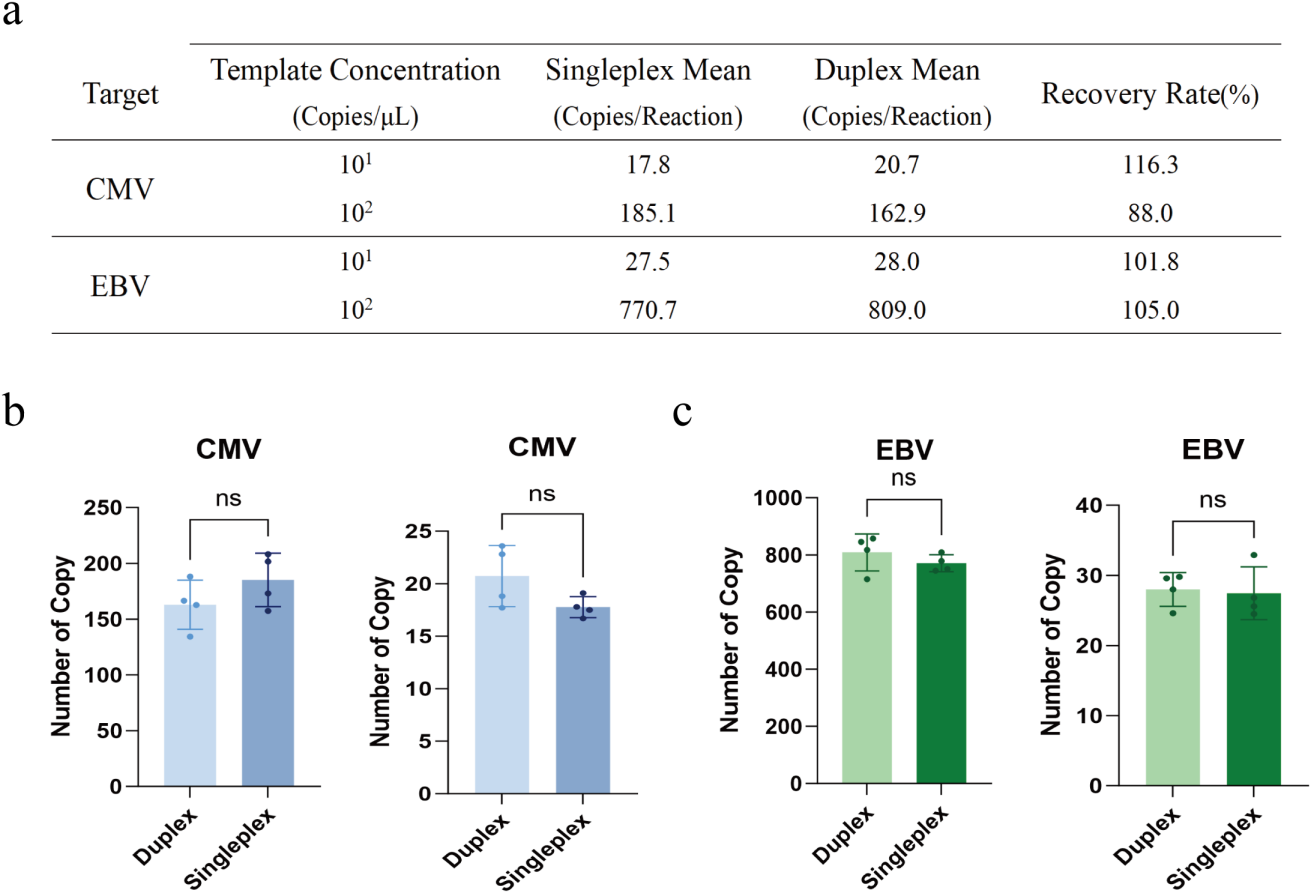
Assessment of competitive inhibition between CMV and EBV targets in the duplex ddPCR system. Two concentrations of inactivated viral DNA standards (10^1^ and 10^2^ copies/μL) were tested to evaluate potential interference between primers and probes in the duplex reaction. **(a)** Summary of mean copy numbers and recovery rates comparing singleplex and duplex detection modes. The recovery rate was calculated as: (Mean Duplex/Mean Singleplex) × 100%.Statistical comparison of quantitative results for **(b)** CMV and **(c)** EBV. In each panel, the left graph corresponds to the medium concentration (10^2^ copies/μL) and the right graph to the low concentration (10^1^ copies/μL). Experiments were performed in quadruplicate (n=4). “no”denotes no statistically significant difference (P>0.05, paired t-test) between the two detection modes.

To ensure the absence of competitive inhibition between primers and probes when detecting CMV and EBV simultaneously in the same reaction tube, inactivated viral DNA standards at two levels, 10^2^ copies/µL and 10^1^ copies/µL, were tested in both singleplex and duplex modes (4 replicates each). At the 10^2^ copies/µL level, paired t-tests showed no statistically significant difference between duplex and singleplex detection results (*p*>0.05); the average recovery rates for CMV and EBV were 88.0% and 105.0%, respectively, showing high consistency. Similarly, at the low concentration level of 10^1^ copies/µL, although the sample size was near the detection limit, statistical analysis confirmed no significant difference in copy numbers between the two modes (*p*>0.05); recovery rates for CMV and EBV were 116.3% and 101.8% (as shown in **Figure 5**). These data indicate that the established duplex ddPCR system has good reaction compatibility, with no obvious competitive inhibition interference between the two targets even under low viral load conditions.

### 3.4 Evaluation of analytical specificity

**Figure 6:**
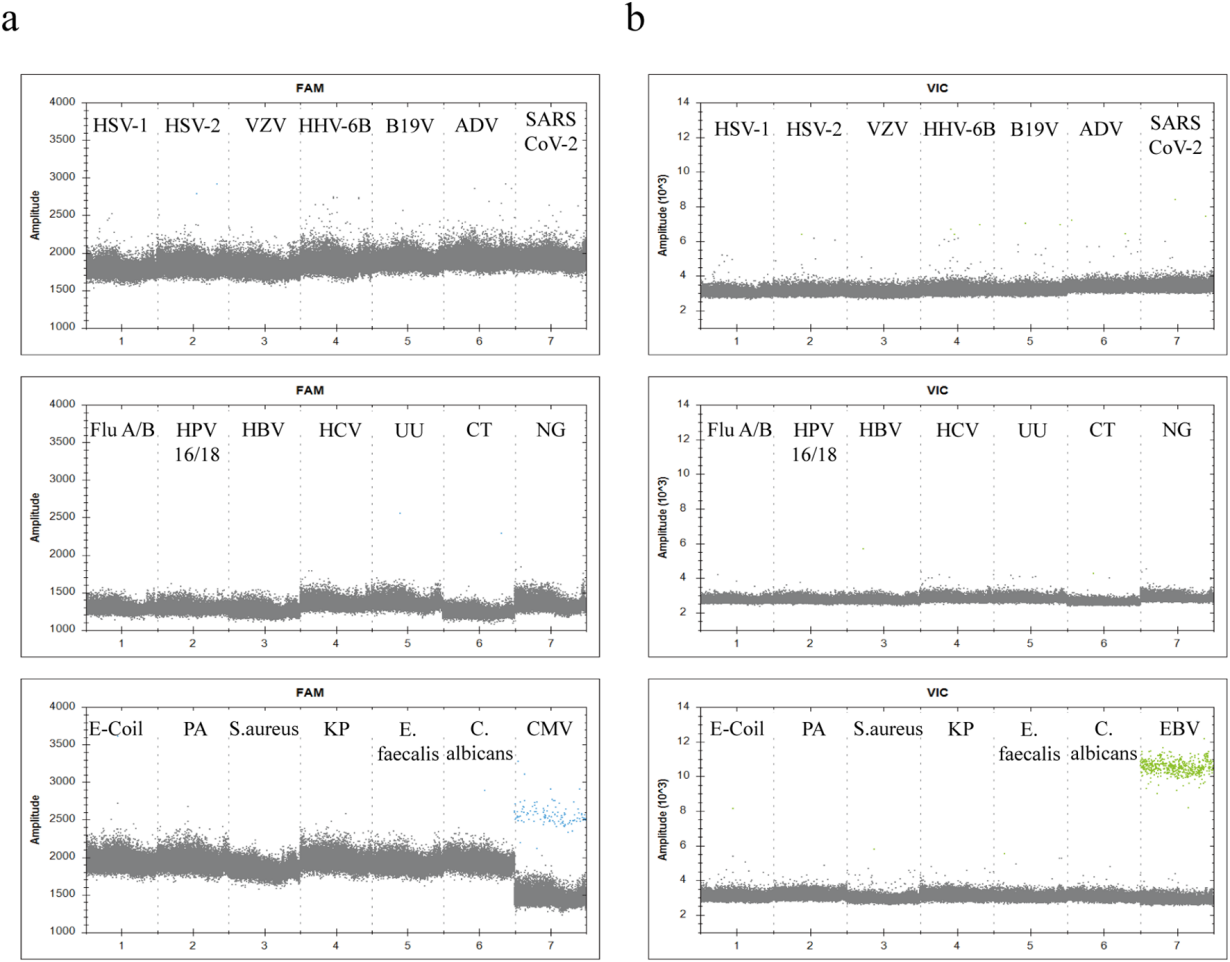
Evaluation of the analytical specificity of the duplex ddPCR assay. The specificity of the duplex ddPCR assay was evaluated against pathogens selected for their genetic homology, similar clinical symptoms, or potential for co-infection with the targets. Genomic DNA or nucleic acid extracts from bacteria, fungi, and viruses were tested. **(a)** 1D amplitude plot for the FAM channel (CMV). **(b)** 1D amplitude plot for the VIC channel (EBV).In both panels, the non-target samples (listed in **Table 1**) resulted in negative droplet clusters corresponding to background signals. Positive droplet clusters were observed only in the final lanes containing inactivated CMV and EBV viral DNA standards, which served as positive controls.

To comprehensively evaluate the analytical specificity of the duplex ddPCR system and exclude cross-reactivity with non-target pathogens, a total of 20 pathogens with homology to CMV/EBV, similar clinical symptoms, or frequent occurrence in bloodstream coinfections were selected for validation (as shown in **Table 1**). Specificity testing results showed that when testing nucleic acids from these non-target pathogens using the duplex ddPCR system, scatter plots for both FAM and VIC channels displayed negative droplet clusters, and concentration readings were all below their respective LODs, with no non-specific amplification signals observed. Positive droplet signals appeared only when CMV or EBV control samples corresponding to specific primers were added to the reaction system (as shown in **Figure 6**). These results confirm that the system possesses good specificity and can effectively resist interference from complex pathogen backgrounds in clinical samples.

**Table 1:** List of non-target pathogens and clinical samples used for analytical specificity evaluation.

Organism	Abbreviation	Sample type	Source
Herpes simplex virus type 1	HSV-1	Positive Clinical Specimen	PUMCH
Herpes simplex virus type 2	HSV-2	Clinical Positive QC	Beijing YouAn Hospital
Varicella-zoster virus	VZV	Positive Clinical Specimen	PUMCH
Human herpesvirus 6B	HHV-6B	Positive Clinical Specimen	NIFDC
Parvovirus B19	B19V	Positive Clinical Specimen	NIFDC
Adenovirus	ADV	Positive Clinical Specimen	PUMCH
Severe acute respiratory syndrome coronavirus 2	SARS-CoV-2	Clinical Positive QC	Beijing YouAn Hospital
Influenza A/B virus	Flu A/B	Clinical Positive QC	Beijing YouAn Hospital
Human papillomavirus type 16/18	HPV-16/18	Clinical Positive QC	Beijing YouAn Hospital
Hepatitis B virus	HBV	Clinical Positive QC	Beijing YouAn Hospital
Hepatitis C virus	HCV	Clinical Positive QC	Beijing YouAn Hospital
Ureaplasma urealyticum	UU	Clinical Positive QC	Beijing YouAn Hospital
Chlamydia trachomatis	CT	Clinical Positive QC	Beijing YouAn Hospital
Neisseria gonorrhoeae	NG	Clinical Positive QC	Beijing YouAn Hospital
Escherichia coli	E. coli	Reference strain (ATCC 25922)	Beijing YouAn Hospital
Pseudomonas aeruginosa	PA	Reference strain (ATCC 27853)	Beijing YouAn Hospital
Staphylococcus aureus	S. aureus	Reference strain (ATCC 29213)	Beijing YouAn Hospital
Klebsiella pneumoniae	KP	Reference strain (ATCC 7006003)	Beijing YouAn Hospital
Enterococcus faecalis	E. faecalis	Reference strain (ATCC 29212)	Beijing YouAn Hospital
Candida albicans	C. albicans	Reference strain (ATCC 29212)	Beijing YouAn Hospital

PUMCH, Peking Union Medical College Hospital; NIFDC, National Institutes for Food and Drug Control; ATCC, American Type Culture Collection; QC, Quality Control.

The specificity of the duplex ddPCR assay was evaluated by testing nucleic acid extracts from the listed organisms to verify the absence of cross-reactivity.

### 3.5 Repeatability and reproducibility of the duplex ddPCR system

To evaluate the precision of the duplex ddPCR system, samples at two concentration levels (nominal 10^2^ and 10^1^ copies/µL) were selected for validation.

Results showed that the system performed stably at both levels. At the high concentration level (nominal 10^2^ copies/µL), the repeatability (intra-assay) results showed measured means of 161.2 copies/reaction for CMV and 803.1 copies/reaction for EBV, with CVs of 11.8% and 7.3%, respectively. At the lower concentration level (nominal 10^1^ copies/µL), the method maintained good repeatability: the measured intra-assay means were 19.7 copies/reaction for CMV and 25.7 copies/reaction for EBV, with CVs of 12.1% and 15.3%, respectively.

Reproducibility (inter-assay) results were consistent, with measured means of 162.0 copies/reaction for CMV and 774.8 copies/reaction for EBV at the high level, and 18.9 copies/reaction for CMV and 25.8 copies/reaction for EBV at the low level (as shown in **Table 2**). Additionally, the stability of the droplet generation process was verified throughout these experiments. The mean number of accepted droplets per reaction consistently exceeded 40,000 (as shown in **Supplementary Table 3**), satisfying the quality control criteria for high-precision digital PCR. This high partition count ensures minimal relative uncertainty and robust statistical power, in accordance with the Digital MIQE Guidelines (2020).

**Table 2:** Evaluation of the intra-assay and inter-assay precision of the duplex ddPCR assay.

Target	Intra-assay			Inter-assay		
	Mean (Copies/Reaction)	SD	CV(%)	Mean (Copies/Reaction)	SD	CV(%)
CMV	161.2	19.0	11.8	162.0	23.8	14.7
	19.7	2.4	12.1	18.9	2.3	12.1
EBV	803.1	58.8	7.3	774.8	60.7	7.8
	25.7	3.9	15.3	25.8	3.6	13.7

SD, Standard Deviation; CV, Coefficient of Variation.

Precision was evaluated using commercial inactivated viral DNA standards at two concentration levels (10^2^ and 10^1^ copies/μL). Intra-assay precision was determined by testing 8 replicates in a single run. Inter-assay precision was calculated based on 8 replicates performed by different operators.

### 3.6 Clinical sample validation and performance comparison between duplex ddPCR and qPCR

To validate the performance of the duplex ddPCR system in actual clinical applications, 117 plasma samples from patients with suspected CMV or EBV infection were collected for analysis. Based on the LOD established in the method validation and the nucleic acid extraction protocol (250 µL plasma input, 80 µL elution), the theoretical detection limit of the ddPCR method for plasma samples was calculated to be 506 copies/mL (CMV) and 416 copies/mL (EBV), which is on the same order of magnitude as the commercial clinical qPCR kit used as a reference (LOD = 500 copies/mL).

**Figure 7:**
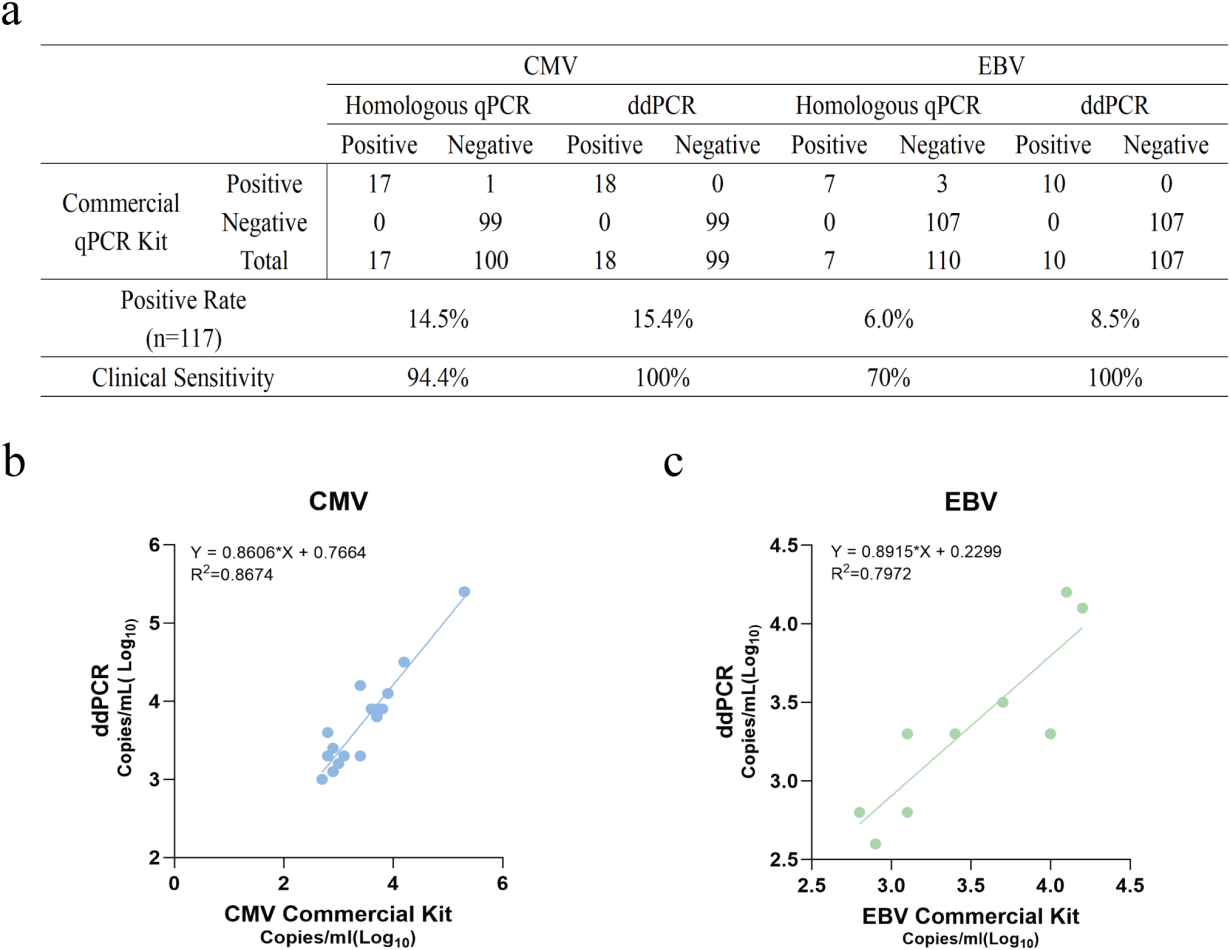
Clinical performance evaluation and method comparison using 117 clinical plasma samples. The performance of the duplex ddPCR assay was compared with a homologous qPCR assay and a commercial qPCR kit (reference standard). **(a)** Comparison of diagnostic results and clinical sensitivity for CMV and EBV. The table summarizes the concordance between the tested methods and the commercial kit, including the number of positive/negative cases, positive rates, and calculated clinical sensitivity. **(b, c)** Linear regression analysis comparing the viral loads quantified by the duplex ddPCR assay and the commercial qPCR kit for **(b)** CMV and **(c)** EBV. Quantitative data were converted to Log10 copies/mL for analysis. The solid lines represent the linear regression fit. The regression equations and coefficients of determination (R^2^) are displayed within the graphs.

Using the commercial kit results as the reference standard, the performance of the homologous qPCR method was first evaluated. Results showed that the homologous qPCR method detected 17 positive samples for CMV, with a clinical sensitivity of 94.4% (17/18) and specificity of 100%; for EBV, it detected 7 positive samples, with a clinical sensitivity of 70.0% (7/10) and specificity of 100%. In contrast, the duplex ddPCR method demonstrated robust detection performance, achieving high concordance with the commercial kit, successfully detecting all positive samples identified by the commercial kit (18 CMV, 10 EBV). This increased the clinical sensitivity for both CMV and EBV detection to 100%, while specificity remained at 100% (as shown in **Figure 7a**). Overall, regarding qualitative determination, the duplex ddPCR method maintained 100% concordance with the commercial reference kit across all 117 samples.

Furthermore, to evaluate the consistency in viral load quantification between the two methods, linear regression analysis was performed on the copy numbers measured by duplex ddPCR and the commercial kit. Analysis showed a strong linear correlation between the quantitative results of the duplex ddPCR method and the commercial kit. The coefficient of determination (R^2^) was 0.8674 for CMV detection and 0.7972 for EBV detection (as shown in **Figures 7b, c**). These results indicate that the established duplex ddPCR system is highly consistent with the commercial kit not only in qualitative detection capability but also shows reliable correlation in quantitative values.

### 3.7 Evaluation of anti-interference capability against endogenous substances

Hyperlipidemia and hyperbilirubinemia are common endogenous interference factors in clinical blood samples, potentially inhibiting PCR amplification efficiency or affecting droplet stability. Results showed that in the hyperlipidemia background, the average recovery rates for CMV and EBV were 94.2% and 89.9%, respectively; in the hyperbilirubinemia background, the average recovery rates were 97.6% and 113%, respectively. All recovery rates fell within the acceptable range of 85%–115% (as shown in **Supplementary Figures 7a-c**). This interval was defined in accordance with the FDA Bioanalytical Method Validation Guidance. While The guidance states that “recovery need not be 100 percent” but must be “consistent”, we adopted the standard accuracy acceptance criteria of ±15% to strictly evaluate this consistency. These results demonstrate that the constructed duplex ddPCR system has strong anti-interference capability and can provide accurate quantitative results in complex clinical plasma matrices even under low viral load conditions.

## 4 Discussion

CMV and EBV are two biologically significant herpesviruses with high prevalence globally. While infections in immunocompetent individuals are typically subclinical or manifest as self-limiting diseases such as infectious mononucleosis, it must be noted that these viruses can still precipitate severe clinical consequences even in this population (Bunchorntavakul and Reddy, 2020; Jhaveri et al., 2022). However, the risks are far more pronounced in immunocompromised populations, including SOT and HSCT recipients as well as HIV-infected individuals. Primary infection or reactivation in these groups often triggers fatal multi-organ invasion, graft rejection, and malignancies such as PTLD. Furthermore, because CMV and EBV infections frequently present with similar clinical symptoms and share overlapping epidemiological characteristics, coinfection or dual reactivation is not only common but also easily confused in clinical practice. Research indicates that such coinfection often exhibits synergistic pathogenicity, significantly reducing patient survival and complicating treatment (Zhou et al., 2021; Li et al., 2022). Regarding clinical diagnosis, although serological testing holds a traditional status, its utility for real-time diagnosis in immunocompromised patients is limited. The “window period” for antibody production can delay diagnosis. The persistence or non-specific appearance of IgM antibodies makes it difficult to accurately distinguish between latent and active infections. More critically, the impaired humoral immunity in immunocompromised patients often leads to delayed or insufficient antibody responses, creating a risk of missed diagnosis. In contrast, molecular testing offers superior sensitivity and specificity, enabling the direct measurement of viral nucleic acid copy numbers to accurately reflect *in vivo* viral replication kinetics. This precise quantitative capability is essential for monitoring antiviral efficacy, assessing disease progression, and guiding preemptive clinical therapy (Dioverti et al., 2016; Natori et al., 2018; Abusalah et al., 2020). As a third-generation PCR technology, ddPCR has been widely applied in the detection and diagnosis of various infectious pathogens, demonstrating better precision, reproducibility, sensitivity, and stability compared to qPCR methods (Kojabad et al., 2021; Wang et al., 2022).

To address the urgent clinical need for precise diagnosis and monitoring of CMV and EBV infections, this study successfully developed and validated a duplex ddPCR assay for the simultaneous quantification of CMV and EBV in plasma, based on the TD-1 droplet digital PCR platform. As a proprietary digital PCR system, this platform has previously demonstrated exceptional application value in fields such as tumor gene mutation detection, infectious diseases, and environmental health monitoring (Li et al., 2021; Ai et al., 2024; Xu et al., 2024). During assay construction, to ensure broad coverage, the highly conserved *UL54* gene of CMV and *EBNA-1* gene of EBV were selected as amplification targets across different viral subtypes. However, the quantitative accuracy of ddPCR relies heavily on the quality of droplet generation and clear separation between droplet populations. Suboptimal amplification efficiency or non-specific amplification can lead to the “rain effect,” thereby interfering with Poisson statistical analysis (Whale et al., 2020). Consequently, this study systematically optimized primer/probe concentrations and annealing temperatures to ensure the system achieved optimal fluorescence amplitude and signal-to-noise ratios. Upon successfully establishing the optimized system, we compared the analytical performance of this duplex ddPCR method with a homologous qPCR assay. Results indicated that although the homologous qPCR method had a higher upper dynamic range limit (10^6^ copies/µL), ddPCR demonstrated significant advantages in the low concentration range. The LODs of ddPCR for CMV and EBV were as low as 7.9 and 6.5 copies/reaction, respectively, which is approximately 1/6 to 1/7 of that of homologous qPCR (53.4 and 45.6 copies/reaction). Although the upper quantitative limit of ddPCR is constrained by the total number of generated droplets, leading to saturation above 10^5^ copies/µL, this is acceptable for clinical applications because the range up to 10^5^ copies/µL is sufficient to meet the monitoring needs for high viral loads (Fang et al., 2023; Rampersad et al., 2024). Indeed, the challenge in clinical viral management lies in sensitively capturing low-level viremia near the threshold and tracking viral replication kinetics during disease progression, rather than precisely stratifying extremely high viral loads. Furthermore, regarding common issues in multiplex PCR such as non-specific hybridization and competition for enzymes or reagents between targets, this study confirmed that even under low viral load conditions, the amplification efficiency of both targets in the duplex system showed no statistically significant difference compared to singleplex modes, ensuring the reliability of coinfection monitoring.

In the clinical performance validation using plasma samples from 117 suspected cases, the duplex ddPCR method showed high concordance with a clinically approved commercial nucleic acid detection kit (LOD = 500 copies/mL), achieving 100% clinical sensitivity and specificity. Notably, four samples (1 CMV and 3 EBV) that were missed by homologous qPCR were successfully detected by ddPCR, further confirming its robustness in detecting low-copy samples. After converting based on plasma nucleic acid extraction volume, the system detection limit of this method is approximately 400–500 copies/mL, which is on the same order of magnitude as currently used commercial clinical kits, fully equipping it for clinical diagnostic needs. Additionally, the system exhibited good tolerance to endogenous interfering substances. Hyperlipidemia and hyperbilirubinemia are common interference factors in clinical blood samples, especially from transplant and liver disease patients, and can cause fluorescence quenching or amplification inhibition in traditional qPCR (Schrader et al., 2012). In our interference experiments, even under simulated high lipid and high bilirubin backgrounds, the recovery rate of low-concentration viral samples remained between 85% and 115%. This anti-interference advantage stems primarily from the “end-point” detection principle of ddPCR. Quantification is based on the frequency of positive droplets rather than amplification kinetics curves, thus effectively circumventing fluorescence quenching or amplification inhibition caused by complex clinical matrices.

Although this study demonstrated the robust performance of the duplex ddPCR method, certain limitations remain to be addressed in future research. First, this study was validated only using clinical plasma samples. However, CMV and EBV can infect multiple organs and tissues, including the central nervous system, lungs, or urinary system. In specific disease states such as encephalitis, pneumonia, or hemorrhagic cystitis, viral loads in cerebrospinal fluid (CSF), bronchoalveolar lavage fluid (BALF), or urine possess unique diagnostic significance. Future studies need to further evaluate the performance of this method in these non-plasma samples. Second, the number of positive clinical samples in this study was relatively small (n=28) due to the retrospective nature of sample collection. While the assay showed 100% concordance with the commercial reference kit, claims of superior clinical sensitivity require further validation in larger, multi-center prospective cohorts. This sample size limitation also restricted our comprehensive evaluation of other potential clinical benefits of the ddPCR method compared to existing commercial options, necessitating further verification in future large-scale clinical studies. Despite the concordance in detection rates, the duplex ddPCR assay offers distinct clinical advantages over the single-plex commercial kit used as a reference, primarily driven by its methodological characteristics. First, unlike the commercial kit which detects CMV and EBV in separate reactions, our assay detects both simultaneously. This duplex design significantly conserves valuable clinical samples (requiring only 5 µL of template for both targets), which is critical for pediatric patients or precious specimen types (e.g., cerebrospinal fluid). It also facilitates the early identification of co-infections and differentiation of viral types in a single workflow. Second, the ddPCR method provides absolute quantification without reliance on standard curves. This eliminates variability associated with calibration standards and amplification efficiency differences, offering a more standardized tool for longitudinal viral load monitoring across different laboratories. Finally, regarding robustness, our data confirmed that the assay maintains accurate quantification even in plasma samples with hyperlipidemia or hyperbilirubinemia, ensuring reliability in complex clinical scenarios, such as liver transplant recipients. Additionally, a consensus on the threshold for initiating antiviral intervention has not yet been reached, and data comparability between different detection platforms is lacking. The high-sensitivity ddPCR platform established in this study can provide more precise absolute quantitative data. Future applications in larger-scale, multi-center prospective cohort studies are needed to elucidate the correlation between viral load and disease progression and to verify the comparability of this method across different laboratories.

In summary, this study successfully established a duplex detection method based on ddPCR technology, achieving simultaneous and precise absolute quantification of CMV and EBV in plasma. With its high sensitivity, specificity, and robust anti-interference capability, it serves as a powerful complement to existing qPCR technologies and serological screening for the early and precise monitoring of CMV/EBV viremia.

## Data Availability

The original contributions presented in the study are included in the article/supplementary material. Further inquiries can be directed to the corresponding authors.
