# Supplementary material for: Development and validation of a duplex droplet digital PCR assay for the simultaneous detection of cytomegalovirus and Epstein-Barr virus in plasma
Source: Front Cell Infect Microbiol. 2026 Mar 19;16:1798127. doi: 10.3389/fcimb.2026.1798127 (PMC13044043; doi:10.3389/fcimb.2026.1798127)
Supplement: Supplementary file 1 [file DataSheet1.docx]

**Development and Validation of a Duplex Droplet Digital PCR Assay for the Simultaneous Detection of Cytomegalovirus and Epstein-Barr Virus in Plasma**

**Supplementary materials**

**Supplementary Table 1.** Sequences of Primers and Probes Used for the Duplex ddPCR Assay.

| **Organism** | **Target Gene** | **Sequence(5’-3’)** | **Amplicon Length** |
| --- | --- | --- | --- |
| **CMV** | UL54-F | ACGAATAGTGTTGCCGTGTC | 87bp |
|  | UL54-R | AAACATAGCCGCCACAGAAC |  |
|  | UL54 Probe | FAM-CATCTCTACCGCCGCCGTGC-BHQ1 |  |
| **EBV** | EBNA-1-F | AACTGCCCTTGCTATTCCAC | 234bp |
|  | EBNA-1-R | TCAAAGCTGCACACAGTCAC |  |
|  | EBNA-1 Probe | VIC-TGGAATGGCCCCTGGACCCG-BHQ1 |  |

Note: F, forward primer; R, reverse primer.

**Supplementary Figure 1.** Maps of the recombinant plasmid.


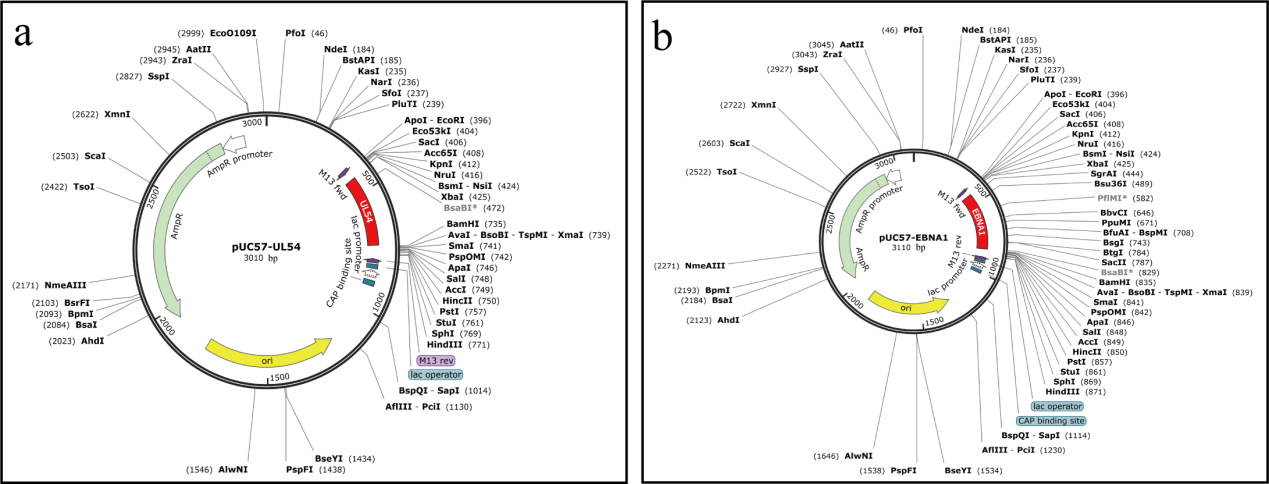


Detailed circular maps of the recombinant plasmids used for evaluating the analytical performance of the duplex ddPCR assay. (a) Map of the pUC57-UL54 plasmid (3010 bp), containing the CMV UL54 gene fragment. (b) Map of the pUC57-EBNA1 plasmid (3110 bp), containing the EBV EBNA-1 gene fragment.

**Supplementary Figure 2.** Agarose gel electrophoresis of PCR amplification products for specificity verification.


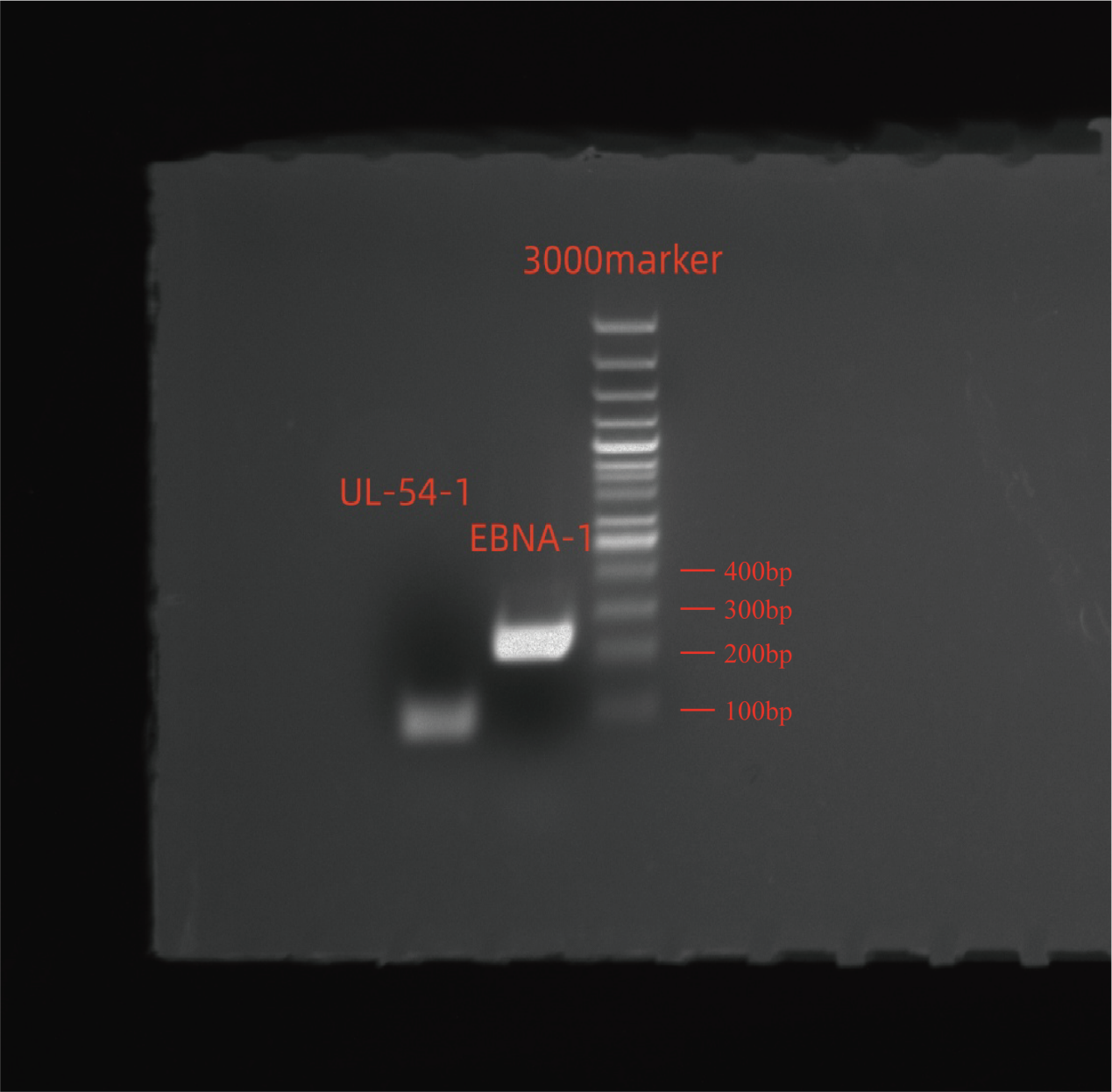


PCR was performed using commercial viral DNA standards as templates to verify the specificity of the designed primers. Left lane (UL54): Amplification product of the CMV UL54 gene showing a specific band at the expected size of 87 bp. Middle lane (EBNA-1): Amplification product of the EBV EBNA-1 gene showing a specific band at the expected size of 234 bp. Right lane (3000marker): DNA molecular weight marker (100–3000 bp range). The single, clear bands indicate high specificity of the primers without non-specific amplification.

**Supplementary Figure 3.** Sanger sequencing validation of the PCR amplification products.


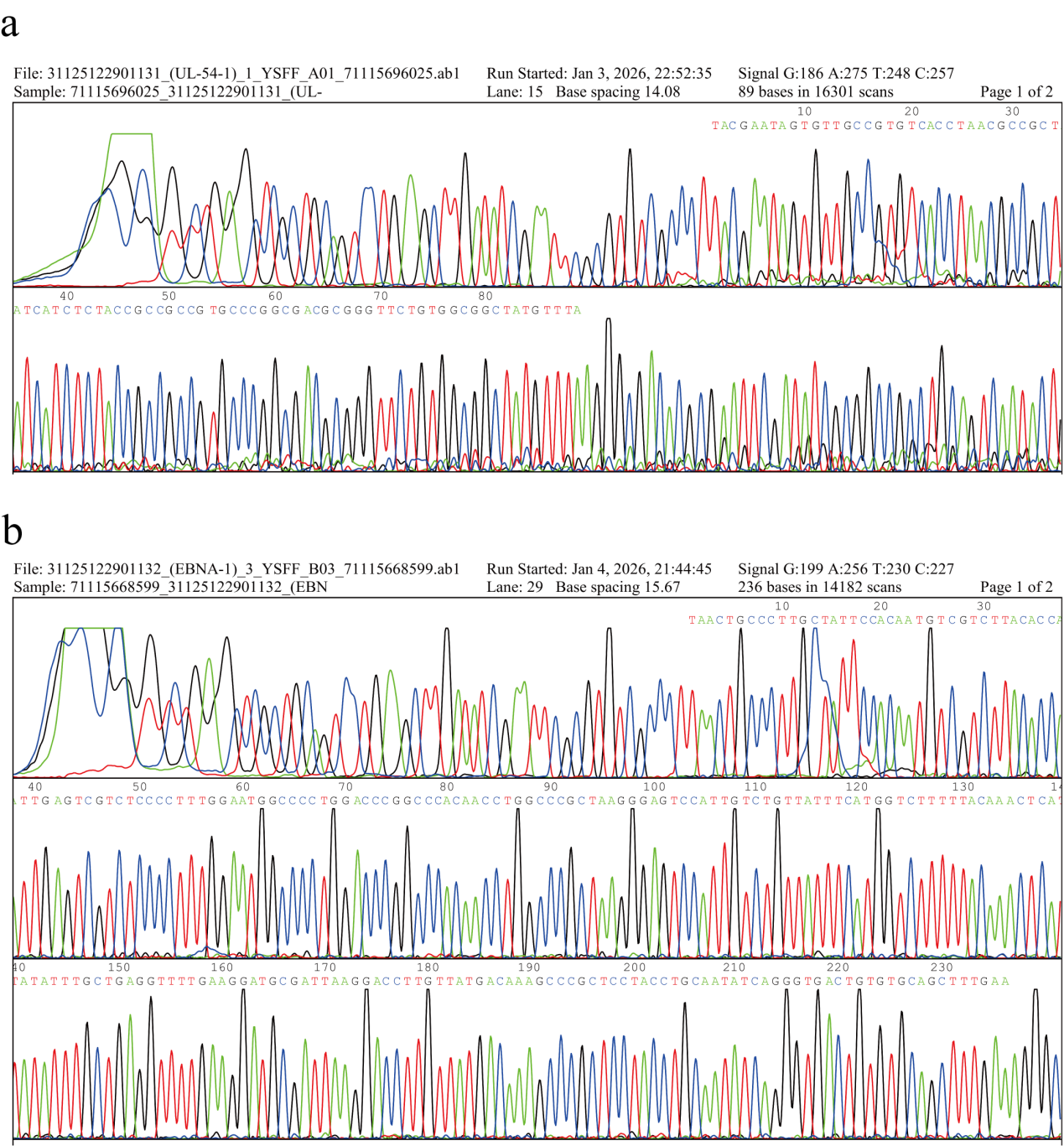


To verify the sequence identity of the amplicons, PCR products were purified and cloned into the pUCm-T vector for Sanger sequencing (Sangon Biotech, Shanghai, China). (a) Sequencing chromatogram of the CMV UL54 gene amplification product. (b) Sequencing chromatogram of the EBV EBNA-1 gene amplification product. The sequencing results showed clear signal peaks and aligned 100% with the corresponding reference sequences in the NCBI GenBank database, confirming the high specificity of the designed primers.

**Reference sequences of the target genes retrieved from NCBI GenBank**

**(Bold text indicates the specific amplicon sequences targeted by the primers and probes)**

>lcl|NC_006273.2_cds_YP_081513.1_1 [gene=UL54] [locus_tag=HHV5wtgp059] [db_xref=GeneID:3077501] [protein=DNA polymerase catalytic subunit] [protein_id=YP_081513.1] [location=complement(78194..81922)] [gbkey=CDS]

1701 AATTCCGTTG CGGCGTGTCA TCTTTGACGG ACAGCAGATC CGTATCTACA

1751 CCTCGCTGCT GGACGAGTGC GCCTGCCGCG ATTTTATCCT GCCCAACCAC

1801 TACAGCAAAG GTACGACGGT GCCCGAA**ACG AATAGTGTTG CCGTGTCACC**

1851 **TAACGCCGCT ATCATCTCTA CCGCCGCCGT GCCCGGCGAC GCGGGTTCTG**

1901 **TGGCGGCTAT GTTT**CAGATG TCGCCGCCCT TGCAATCTGC GCCGTCCAGT

1951 CAGGACGGCG TTTCACCCGG CTCCGGCAGT AACAGTAGTA GCAGCGTCGG

>lcl|NC_007605.1_cds_YP_401677.1_1 [gene=EBNA-1] [locus_tag=HHV4_EBNA-1.2] [db_xref=UniProtKB/TrEMBL:Q777E1] [protein=nuclear antigen EBNA-1] [protein_id=YP_401677.1] [location=95662..97587] [gbkey=CDS]

1501 GGAACTTGGG TCGCCGGTGT GTTCGTATAT GGAGGTAGTA AGACCTCCCT

1551 TTACAACCTA AGGCGAGG**AA CTGCCCTTGC TATTCCACAA TGTCGTCTTA**

1601 **CACCATTGAG TCGTCTCCCC TTTGGAATGG CCCCTGGACC CGGCCCACAA**

1651 **CCTGGCCCGC TAAGGGAGTC CATTGTCTGT TATTTCATGG TCTTTTTACA**

1701 **AACTCATATA TTTGCTGAGG TTTTGAAGGA TGCGATTAAG GACCTTGTTA**

1751 **TGACAAAGCC CGCTCCTACC TGCAATATCA GGGTGACTGT GTGCAGCTTT**

1801 **GA**CGATGGAG TAGATTTGCC TCCCTGGTTT CCACCTATGG TGGAAGGGGC

**Supplementary Figure 4.** Optimization of primer and probe concentrations for the duplex ddPCR assay.


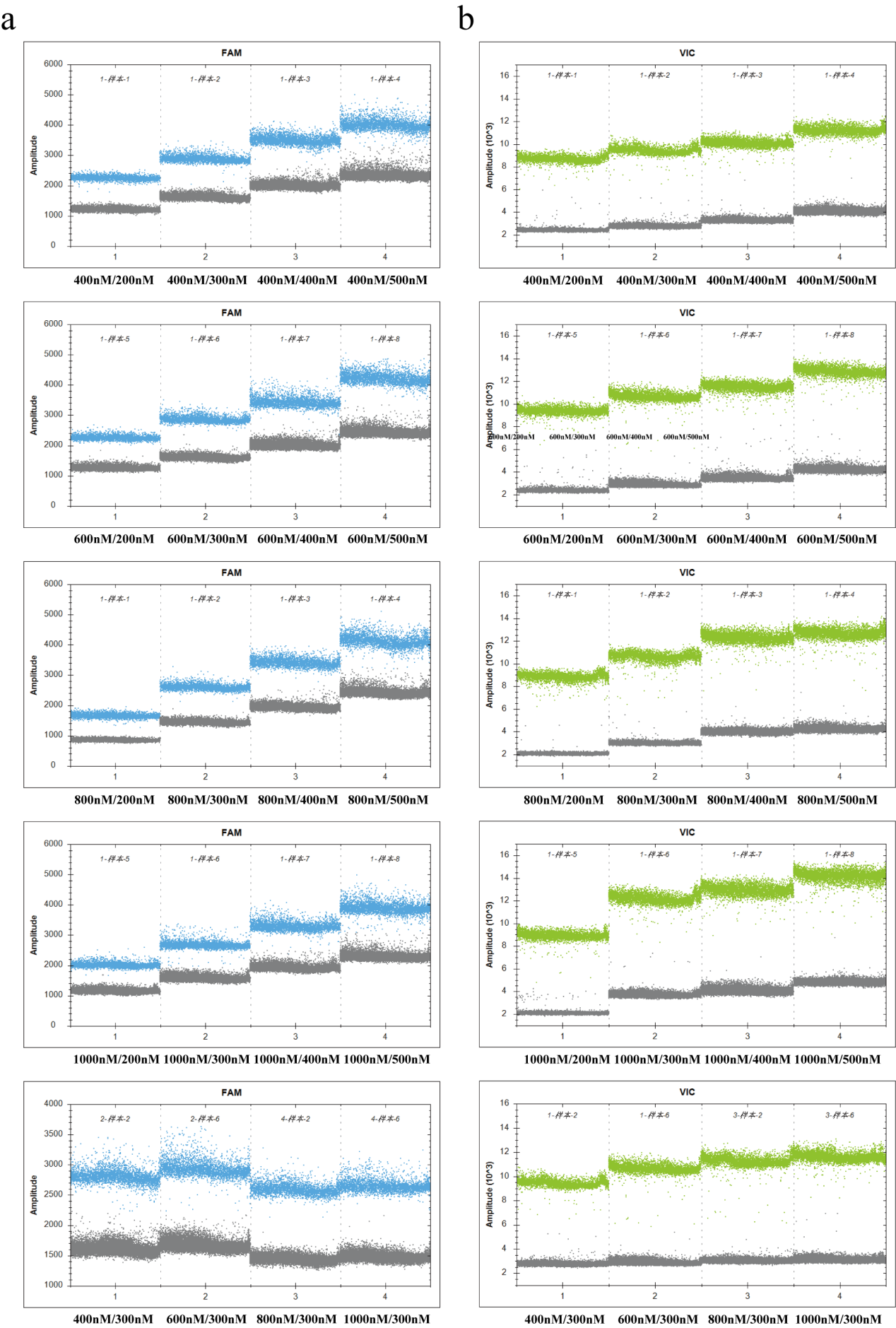


The fluorescence amplitude and cluster separation were evaluated under various concentration combinations to determine the optimal reaction conditions.(a) 1D amplitude plots for the FAM channel (CMV).(b) 1D amplitude plots for the VIC channel (EBV).In each column, the top four rows display a matrix of primer concentrations (400, 600, 800, and 1000 nM) paired with varying probe concentrations (200, 300, 400, and 500 nM). The x-axis labels denote the concentration ratio as “Primer Concentration / Probe Concentration”. The bottom row illustrates the final optimization step, comparing increasing primer concentrations (400–1000 nM) while the probe concentration was fixed at 300 nM. The combination of 800 nM primers and 300 nM probes exhibited the highest fluorescence amplitude and optimal separation for both targets.

**Supplementary Figure 5.** Determination of the Limit of Blank (LOB) for the duplex ddPCR assay.


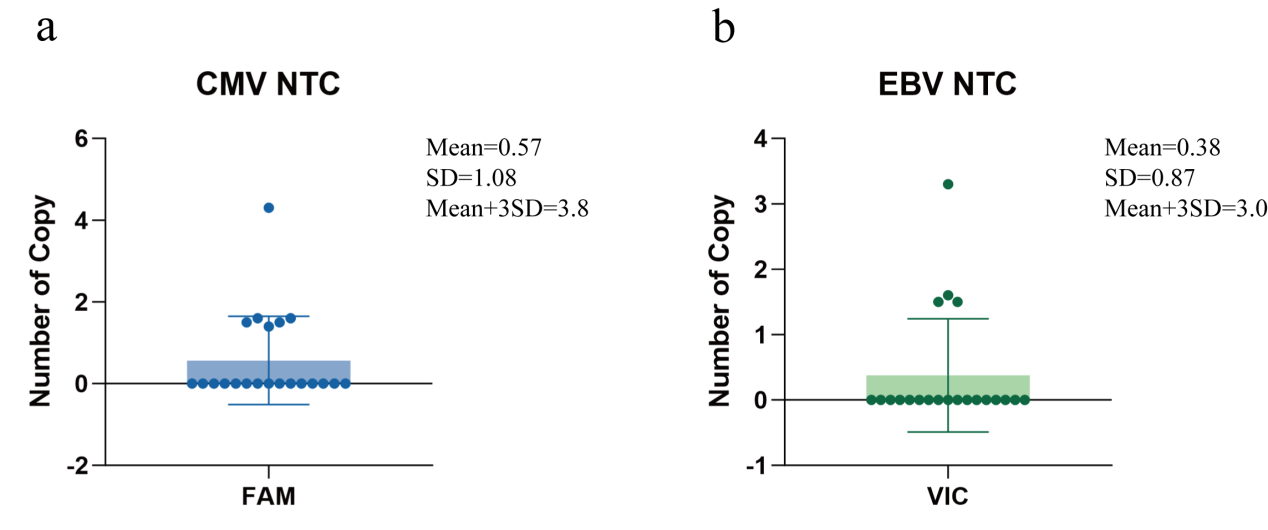


Twenty replicates of non-template controls (NTC) were analyzed to establish the positive determination thresholds. (a) Scatter plot of background signals for CMV (FAM channel). (b) Scatter plot of background signals for EBV (VIC channel). The scatter plots display individual data points for each replicate. The Limit of Blank (LOB) was calculated using the formula Mean + 3×SD. Based on this calculation, the positive cutoff values were set at 3.8 copies/reaction for CMV and 3.0 copies/reaction for EBV.

**Supplementary Table 2.**Comparison of the Limit of Quantification (LOQ) for CMV and EBV between the duplex ddPCR and qPCR assays

| **Organism** | **Method** | **Mean(Copies/Reaction)** | **CV(%)** |
| --- | --- | --- | --- |
| **CMV** | ddPCR | 67.3 | 17.2 |
|  |  | 29.5 | 18.5 |
|  |  | 16.0 | 16.6 |
|  |  | **9.9** | 19.1 |
|  |  | 4.4 | 51.4 |
|  | qPCR | 3839.0 | 14.9 |
|  |  | 407.9 | 12.1 |
|  |  | 208.7 | 13.4 |
|  |  | 105.1 | 12.6 |
|  |  | **57.0** | 14.5 |
|  |  | 35.8 | 30.0 |
| **EBV** | ddPCR | 64.2 | 15.7 |
|  |  | 36.5 | 16.2 |
|  |  | 18.5 | 18.4 |
|  |  | **10.8** | 16.8 |
|  |  | 5.6 | 48.5 |
|  | qPCR | 1082.0 | 3.8 |
|  |  | 470.0 | 6.2 |
|  |  | 265.2 | 7.6 |
|  |  | 85.4 | 10.4 |
|  |  | **46.0** | 16.8 |
|  |  | 15.3 | 28.7 |

Note: Serial dilutions of standard controls were tested in 20 replicates to determine the LOQ. The LOQ was defined as the lowest concentration at which the coefficient of variation (CV) of the measured copy numbers remained below 20%. The determined LOQ values for each method are highlighted in bold.

**Supplementary Table 3.** Summary of Droplet Generation Statistics for Reproducibility Assessment

| **Target Organism** | **Concentration Level**  **(copies/µL)** | **Replicates**  **(n)** | **Mean Total Droplets**  **(per reaction)** | **Standard Deviation (SD)** | **CV (%)** |
| --- | --- | --- | --- | --- | --- |
| CMV | 161.2 | 16 | 42234.3 | 2954.3 | 6.99 |
|  | 19.7 | 16 | 44909.1 | 4682.0 | 10.40 |
| EBV | 803.1 | 16 | 44566.1 | 2221.0 | 4.98 |
|  | 25.7 | 16 | 45624.3 | 4546.0 | 9.96 |

The table summarizes the technical performance of droplet generation during the inter-assay precision study. For each target (CMV and EBV) at both concentration levels (Medium and Low), the mean number of accepted droplets per reaction, standard deviation (SD), and coefficient of variation (CV) were calculated from 16 replicates. The mean droplet count for all reactions consistently exceeded 40,000, significantly surpassing the minimum requirement of 10,000 partitions recommended by the Digital MIQE Guidelines (2020), thereby ensuring robust Poisson statistical analysis.

**Supplementary Text 1.** Technical Details of Droplet Analysis and Quality Control.

**Thresholding and Analysis Software:**

Threshold determination was performed automatically using the TargetingOne dPCR Analysis software. The software employs a clustering algorithm based on the Poisson distribution to differentiate positive from negative partitions, enabling the accurate calculation of absolute nucleic acid copy numbers.

**Droplet Acceptance Criteria and Statistical Robustness:**

A strict quality control workflow was established for droplet generation. As detailed in Supplementary Table 3, the mean number of accepted droplets per reaction consistently exceeded 40,000. This threshold significantly surpasses the minimum requirement of 10,000 partitions recommended by the Digital MIQE Guidelines Update (2020) (Whale et al., 2020). As illustrated in Figure 1 of the guidelines, achieving a partition number >10,000 significantly reduces relative uncertainty (to <5% across the majority of the dynamic range) and ensures robust Poisson statistical modeling.

**Rain Drop Handling and Classification:**

To minimize “rain” (droplets with intermediate fluorescence), thermal cycling conditions were optimized during the assay development phase. In the final data analysis, a conservative classification rule was applied based on the Limit of Blank (LOB) principle. Ambiguous droplets falling between the negative and positive clusters were generally classified as negative to prevent false positive reporting. Specifically, if the absolute copy number calculated including ambiguous signals fell below the established LOB threshold, the sample was reported as negative.

**Supplementary Figure 6.** Representative droplet amplitude plots of a dual-positive clinical plasma sample demonstrating robust separation efficiency.


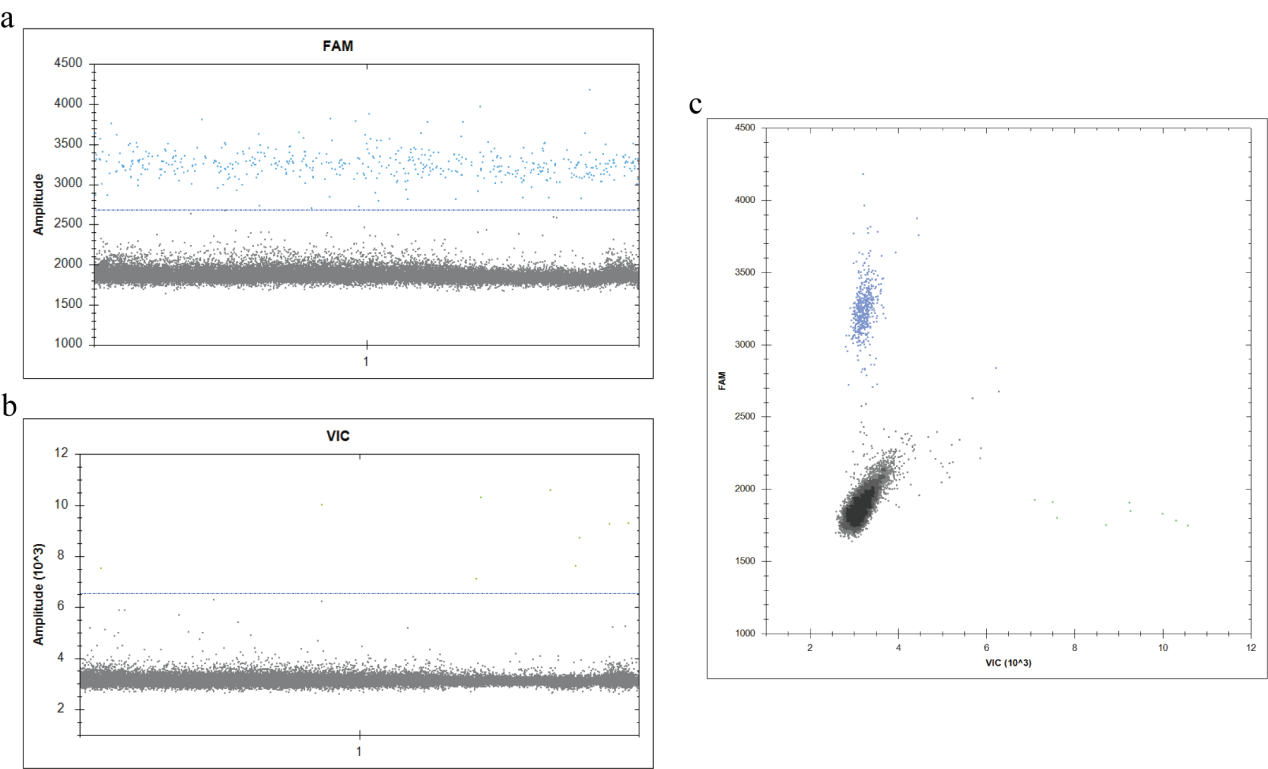


(a) 1D amplitude plot in the FAM channel (CMV), showing a clear distinction between the negative droplet cluster (grey) and the positive droplet cluster (blue). (b) 1D amplitude plot in the VIC channel (EBV), displaying distinct separation between negative (grey) and positive (green) droplets. (c) 2D scatter plot of the same clinical sample, illustrating orthogonal separation of the four droplet populations (negative, CMV-positive only, EBV-positive only, and double-positive). Despite the complex clinical plasma matrix, the assay maintains a clear threshold with minimal intermediate “rain,” ensuring accurate quantification.

**Supplementary Figure 7.** Evaluation of anti-interference capability against endogenous substances in clinical plasma.


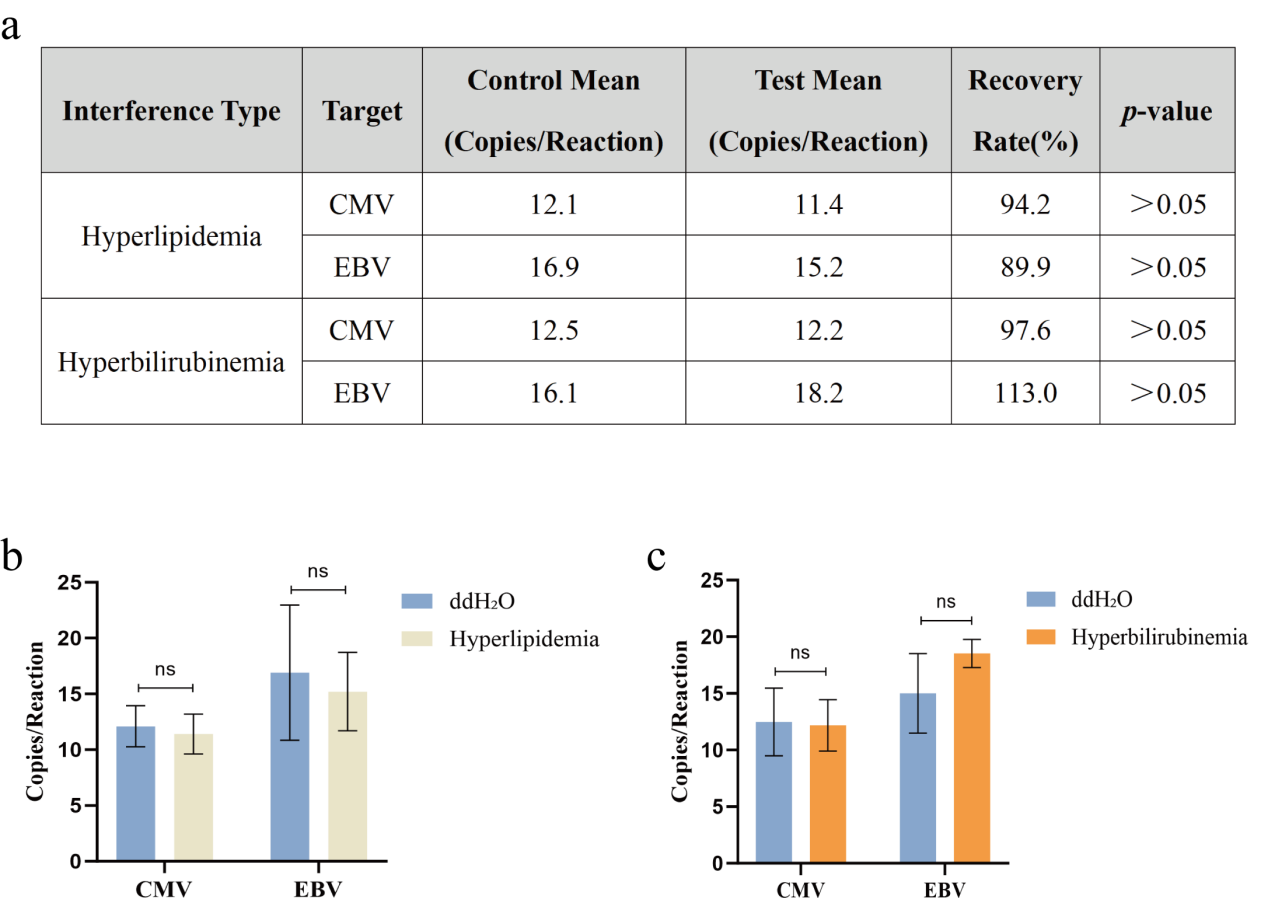


The resilience of the duplex ddPCR assay was tested against hyperlipidemia and hyperbilirubinemia backgrounds. Inactivated viral DNA standards were spiked into the clinical matrices or deionized water (ddH_2_O, control) at concentrations near the LOD.

(a) Summary of mean copy numbers and recovery rates. The recovery rate was calculated as: (Test Mean / Control Mean) × 100%.

(b, c) Statistical comparison of quantitative results between the control group and the interference groups: (b) Hyperlipidemia and (c) Hyperbilirubinemia. Bars represent the mean copy number ± SD from four replicates (n=4). “ns” indicates no statistically significant difference (*p*>0.05, paired t-test).
